# Repetitive somatic embryogenesis induced cytological and proteomic changes in embryogenic lines of *Pseudotsuga menziesii* [Mirb.]

**DOI:** 10.1186/s12870-018-1337-y

**Published:** 2018-08-10

**Authors:** Florian Gautier, Kateřina Eliášová, Jean-Charles Leplé, Zuzana Vondráková, Anne-Marie Lomenech, Claire Le Metté, Philippe Label, Guy Costa, Jean-François Trontin, Caroline Teyssier, Marie-Anne Lelu-Walter

**Affiliations:** 1 BioForA, INRA, ONF, F-45075 Orléans, France; 2 SylvaLIM, University Limoges, F-78060 Limoges, France; 3 Institute of Experimental Botany of the Czech Academy of Sciences, Rozvojová 263, 165 02 Praha, 6-Lysolaje Czech Republic; 4 BIOGECO, INRA, University Bordeaux, F-33610 Cestas, France; 5 Plateforme Protéome, Centre de Génomique Fonctionnelle, University Bordeaux, F-33000 Bordeaux, France; 6 University Clermont Auvergne, INRA, PIAF, F-63000 Clermont–Ferrand, France; 7 Pôle Biotechnologie et Sylviculture Avancée, FCBA, Campus Forêt-Bois de Pierroton, F-33610 Cestas, France

**Keywords:** Douglas-fir, Embryogenic potential, Proliferation, Proteomic, Cytology, Plant growth regulators

## Abstract

**Background:**

To explore poorly understood differences between primary and subsequent somatic embryogenic lines of plants, we induced secondary (2^ry^) and tertiary (3^ry^) lines from cotyledonary somatic embryos (SEs) of two Douglas-fir genotypes: SD4 and TD17. The 2^ry^ lines exhibited significantly higher embryogenic potential (SE yields) than the 1^ry^ lines initiated from zygotic embryos (SD4, 2155 vs 477; TD17, 240 vs 29 g^− 1^ f.w.). Moreover, we observed similar differences in yield between 2^ry^ and 3^ry^ lines of SD4 (2400 vs 3921 g^− 1^ f.w.). To elucidate reasons for differences in embryogenic potential induced by repetitive somatic embryogenesis we then compared 2^ry^ vs 1^ry^ and 2^ry^ vs 3^ry^ lines at histo-cytological (using LC-MS/MS) and proteomic levels.

**Results:**

Repetitive somatic embryogenesis dramatically improved the proliferating lines’ cellular organization (genotype SD4’s most strongly). Frequencies of singulated, bipolar SEs and compact polyembryogenic centers with elongated suspensors and apparently cleavable embryonal heads increased in 2^ry^ and (even more) 3^ry^ lines. Among 2300–2500 identified proteins, 162 and 228 were classified significantly differentially expressed between 2^ry^ vs 1^ry^ and 3^ry^ vs 2^ry^ lines, respectively, with special emphasis on “Proteolysis” and “Catabolic process” Gene Ontology categories. Strikingly, most of the significant proteins (> 70%) were down-regulated in 2^ry^ relative to 1^ry^ lines, but up-regulated in 3^ry^ relative to 2^ry^ lines, revealing a down-up pattern of expression. GO category enrichment analyses highlighted the opposite adjustments of global protein patterns, particularly for processes involved in chitin catabolism, lignin and L-phenylalanine metabolism, phenylpropanoid biosynthesis, oxidation-reduction, and response to karrikin. Sub-Network Enrichment Analyses highlighted interactions between significant proteins and both plant growth regulators and secondary metabolites after first (especially jasmonic acid, flavonoids) and second (especially salicylic acid, abscisic acid, lignin) embryogenesis cycles. Protein networks established after each induction affected the same “Plant development” and “Defense response” biological processes, but most strongly after the third cycle, which could explain the top embryogenic performance of 3^ry^ lines.

**Conclusions:**

This first report of cellular and molecular changes after repetitive somatic embryogenesis in conifers shows that each cycle enhanced the structure and singularization of EMs through modulation of growth regulator pathways, thereby improving the lines’ embryogenic status.

**Electronic supplementary material:**

The online version of this article (10.1186/s12870-018-1337-y) contains supplementary material, which is available to authorized users.

## Background

Plant somatic embryogenesis is the generation of embryos from vegetative cells, usually in vitro. Whenever possible, it is the preferred option for true-to-type vegetative propagation of selected genotypes as both apical and root embryonic meristems are delineated early during establishment of the embryo body plan. Thus, in contrast to other vegetative propagation technologies, there is no need for adventitious organogenesis. In recent years, significant advances have been made in the development of techniques to improve somatic embryogenesis of increasing numbers of tree species, from initiation of embryogenic cultures to maturation of high-quality somatic embryos (SEs). Such progress towards large-scale production of vigorous somatic seedlings has been reported for both hardwood [1, 2] and softwood (mostly coniferous) species (reviewed in [3]). Somatic embryogenesis techniques for propagating Douglas-fir (*Pseudotsuga menziesii* (Mirb) Franco), a productive conifer species of the *Pinaceae* family with globally appreciated wood quality, have been under development for more than 30 years [4]. There is a wealth of patented methods, but some recent improvements for steps from initiation to efficient production of somatic seedlings have just been made publicly available ([5, 6], and references therein).

Somatic embryogenesis is considered a promising biotechnology for large-scale clonal propagation of forest trees, due to the high multiplication rates it can provide [7, 8]. Moreover, embryogenic cultures are amenable to both cryogenic storage for long-term preservation of genetic resources [3, 7] and genetic engineering (including genome editing) for functional characterization of genes expressed during embryogenesis [9]. Somatic embryogenesis is also a convenient experimental model system for studying embryo development [9]. The process includes well-characterized developmental stages and pathways that are mostly similar between SEs and reference zygotic embryos [10], as shown recently in conifers at levels ranging from the molecular (in hybrid larch [11]) to morphological (in maritime pine [12]).

Once somatic embryogenesis has been initiated, embryogenic cultures are proliferated to sustain new embryo formation. In angiosperms, embryogenic potential is maintained during a continuous process of repetitive, secondary (2^ry^) embryogenesis, either directly from primary (1^ry^) embryos in culture or indirectly from various cell aggregates such as proembryogenic masses or nodular calli developing from 1^ry^ SEs [13, 14]. This process typically results in clusters of new SEs that are detachable, to varying degrees, from the previous embryo explants.

Embryogenic cultures of gymnosperms proliferate as embryonal masses (EMs), i.e. clusters of multiple attached SEs that become interspersed with singulated SEs at an early stage of late embryogeny [4, 15]. EMs typically have a whitish to translucent appearance and may have a granular to spiky morphotype due to early embryos protruding at their surface. These immature SEs are typically bipolar structures composed of an apical embryonal head (composed of dense, meristematic cells) tightly connected to a basal suspensor tissue (long, vacuolated cells). Proliferation of EMs is thought to mainly result from high cleavage ability of immature, early SEs. This process is known as cleavage polyembryony in gymnosperms, and can naturally occur in seeds of some genera (e.g. *Pinus* species). It is still unclear if cleavage polyembryony is the only process involved in early SEs’ proliferation.

SE clusters of some conifer species, such as Douglas-fir, develop into polyembryogenic center of various sizes, putatively through continuous but incomplete cleavage polyembryony (somatic polyembryogenesis) and /or de novo somatic embryogenesis from proliferating early SEs [6]. In addition, some Douglas-fir lines of proliferating EMs contain both immature SEs and clusters of non-embryogenic cells (NECs) [5, 16]. The occurrence of viable NECs interspersed with early SEs is apparently a characteristic feature of Douglas-fir EMs that has not been clearly documented in other conifer species ([5], Eliášová and Lelu-Walter, personal communication). Subsequent transition from early SEs to cotyledonary SEs is stimulated when EMs are exposed to specific maturation conditions. Usual requirements for this transition, *inter alia* for Douglas-fir cultures [6], are supplementation of the medium with abscisic acid (ABA) in conjunction with increases in osmotic pressure (using a solution with high carbohydrate concentration, such as 0.2 M sucrose), and/or reduction of the water potential using high molecular weight polymers (such as 4000 Da polyethylene glycol, PEG 4000) or physically reducing water availability for the cultured cells by increased the medium’s gel strength.

Somatic embryogenesis of angiosperm tree species can be initiated not only from juvenile material, but also from tissues obtained from mature trees, e.g. up to 100-years-old in *Quercus* spp. trees [17] and even 700-year-old in *Kalopanax septemlobus* trees [18]. Although direct initiation from old tree explants is difficult and requires preliminary conditioning through *in vitro* and/or rejuvenation techniques (establishment of axillary shoot cultures, grafting, etc.), the embryogenic capacity of initiated lines can usually be maintained for years by repetitive somatic embryogenesis. Moreover, with material of many angiosperm species, 2^ry^ embryogenesis is much more efficient than 1^ry^ embryogenesis [19] (and references therein). In contrast, initiating somatic embryogenesis from zygotic explant material of conifers older than zygotic embryos or very young plants is still problematic [20, 21] (and references therein). However, it has been known for 25 years that explants derived from somatic material of *Picea abies* is much more responsive to somatic embryogenesis induction treatment than material derived from zygotic embryos (one-month-old plantlets [22]). Accordingly, the ability to initiate 2^ry^ somatic embryogenesis has been observed in cotyledonary SEs and somatic seedlings of various coniferous species, including *Picea glauca* (up to 10-year-old trees, [23]), *Picea abies* (up to 3-year-old plants, [22, 24, 25]), *Picea mariana* (cotyledonary SEs, [26]), *Larix* x *leptoeuropaea* (up to germinated SEs, [27, 28]), *Abies numidica* (cotyledonary SEs, [29, 30]), *Pinus pinaster* (up to germinating SEs, [31]) and more recently Douglas-fir (cotyledonary SEs, [6]). In most species, 2^ry^ somatic embryogenesis can be initiated at quite high frequency from cotyledonary SEs.

In conifers, 2^ry^ somatic embryogenesis has numerous potential applications as it offers a potential means to obtain “immortal” embryogenic lines [32]. Stable lines are extremely attractive for long-term fundamental studies of plant embryo development, as their use reduces severe experimental constraints, such as culture aging [19, 32]. Secondary somatic embryogenesis can also be used for restoring the embryogenic capacity of aging/failing lines that have diminishing maturation ability and/or are producing abnormal or poor-quality embryos. Another particularly interesting practical feature is that 2^ry^ somatic embryogenesis could be useful for improving the embryogenic potential of some species’ embryogenic lines. For example, 2^ry^ EMs are reportedly more productive than 1^ry^ cultures of some embryogenic lines of hybrid larch [27] and maritime pine [31]. Similarly, 2^ry^ lines obtained from recalcitrant genotypes of Douglas-fir with low embryogenic potential (< 500 SEs g^− 1^ EMs f.w.) have been found to be significantly more productive than 1^ry^ lines [6].

The reasons for such differences in embryogenic potential between 2^ry^ and 1^ry^ lines of conifers are largely unknown, and have only been previously examined in one study focused on *Pinus pinaster* [31]. Clearly, detailed knowledge of cytological and molecular events that occur in proliferating embryogenic lines during repetitive somatic embryogenesis cycles are required. In the study presented here we attempted to acquire such knowledge, and test the hypothesis that during 2^ry^ somatic embryogenesis selective processes may occur that promote development of embryogenic cells and EMs with high capacities to proliferate and regenerate cotyledonary SEs.

In contrast to macromorphological observations, cytological features of EMs during proliferation are reliable indicators of embryogenic lines’ ability to produce cotyledonary SEs. In Norway spruce, only EMs showing immature SEs with a dense embryonal head clearly separated from a well-defined suspensor region can reportedly develop further into cotyledonary SEs [33], at least under conditions applied in the cited study. In pine species, embryogenic potential is reduced in over-propagated EMs due to aging effects (reviewed in [8]). Such poor performance at the maturation step was shown to be associated with substantial progressive changes in cellular organization during proliferation in *Pinus pinaster*, resulting in reduced frequencies of immature SEs capable of completing the last stages of late embryogenesis [34]. In both maritime pine and Douglas-fir, proliferation of EMs in the presence of maltose as the main carbon source can greatly improve the key cytological features of immature SEs [5, 35].

Recently, the development of novel, high-resolution proteomic methods has offered opportunities for both untargeted qualitative proteome coverage and quantitative measurement of proteins involved in plant development. These proteomic analyses have already improved understanding of the metabolic and signaling pathways involved in plant somatic embryogenesis [9, 36, 37]. In conifers, significant changes in protein expression during early and late somatic or zygotic embryogenesis, have been reported as reviewed in [21], in various species including members of *Pinaceae* family, such as *Picea glauca* [38], *Picea abies* [39], *Larix* spp. [40–42], *Pinus massoniana* [43] and *Pinus pinaster* [12, 44].

The main objective in the work presented here was to study cellular and proteomic changes induced by repetitive somatic embryogenesis in Douglas-fir. For this purpose, somatic embryogenesis was induced in cotyledonary SEs to obtain 2^ry^ lines, and 2^ry^ EMs were compared to 1^ry^ EMs in terms of embryogenic potential, cytologicaly and protein patterns during proliferation. To deepen understanding of repetitive somatic embryogenesis, tertiary (3^ry^) lines were produced from cotyledonary SEs of 2^ry^ lines via a third cycle of somatic embryogenesis, and 3^ry^ EMs were compared to 2^ry^ EMs. This is the first report of two successive cycles of repetitive somatic embryogenesis in conifers. We first compared 2^ry^ EMs to 1^ry^ EMs, then 2^ry^ to 3^ry^ EMs. However, cells of 3^ry^ EMs do not directly stems from 1^ry^ EMs, and many subcultural steps involving physiological aging of the tissues could occur between them. Thus, we did not compare 1^ry^ and 3^ry^ EMs to avoid possible complexities irrelevant to aims of this study.

We obtained the first evidence of cytological and proteomic changes in proliferating EMs of Douglas-fir with increasing embryogenic potential following repetitive somatic embryogenesis. Interestingly, the proteomic analysis further revealed different sets of proteins that are significantly differentially expressed between 2^ry^ and 1^ry^ EMs, and between 2^ry^ and 3^ry^ EMs (hereafter “significant” proteins), suggesting that each cycle of repetitive somatic embryogenesis promotes substantial genome-wide rearrangement of gene expression patterns. In addition, Sub-Network Enrichment Analysis (SNEA) was performed to elucidate the functions and interactions of identified proteins, and the results show that this novel approach for studying conifer somatic embryogenesis can yield valuable information.

## Methods

### Plant material

*Pseudotsuga menziesii* trees involved in this work are descendants of trees with provenances in North Bend (genotypes 4455 and 4456) or Enumclaw (4466 and 4477), Washington (USA). They were used as parental trees to perform the following control crosses at INRA (Orléans, France): 4455 × 4466 and 4456 × 4477. Somatic embryogenesis was induced from seed explants (isolated immature zygotic embryos at the pre-cotyledonary developmental stage) following published methodology [5]. Primary embryogenic lines SD4 (4456 × 4477) and TD17 (4455 × 4466) were initiated in 2011 and 2012, respectively [6].

### Methods

#### Proliferation of embryonal masses

EMs were sub-cultured in clumps every 2 weeks on Glitz proliferation medium, consisting of modified Litvay medium [45, 46] supplemented with 4.5 μM 2,4-D (2,4-dichlorophenoxyacetic acid), 2.2 μM BA (6-benzyladenine) and 0.087 M maltose, solidified with 4 g L^− 1^ gellan gum. When necessary, EMs were cultured as a thin layer dispersed on a filter paper disc (300 mg f.w. per filter) to promote proliferation, as previously described [6]. The pH of each medium was adjusted to 5.8 before autoclaving.

#### Repetitive somatic embryogenesis (Fig. 1)

Two cycles of repetitive somatic embryogenesis were performed, following published protocols [6]. Briefly, for the first induction cycle, 6- to 11-week-old cotyledonary SEs (see maturation section) regenerated from 1^ry^ embryogenic lines SD4 and TD17 were isolated and transferred to Glitz initiation medium supplemented with 4.5 μM 2,4-D, 4.4 μM BA and 0.087 M sucrose, solidified with 4 g.L^− 1^ gellan gum. Each 2^ry^ EM, initiated from a single SE, was then subcultured as described above for proliferation. We obtained 2^ry^ embryogenic lines designated SD4–2, SD4–6 and SD4–8 from the 1^ry^ line SD4, and a 2^ry^ line designated TD17–1 from the 1^ry^ TD17 line. For the second induction cycle, cotyledonary SEs obtained from 2^ry^ line SD4–8 were similarly used as explants to initiate 3^ry^ lines, designated SD4–8-1, SD4–8-2 and SD4–8-3.

**Fig. 1:**
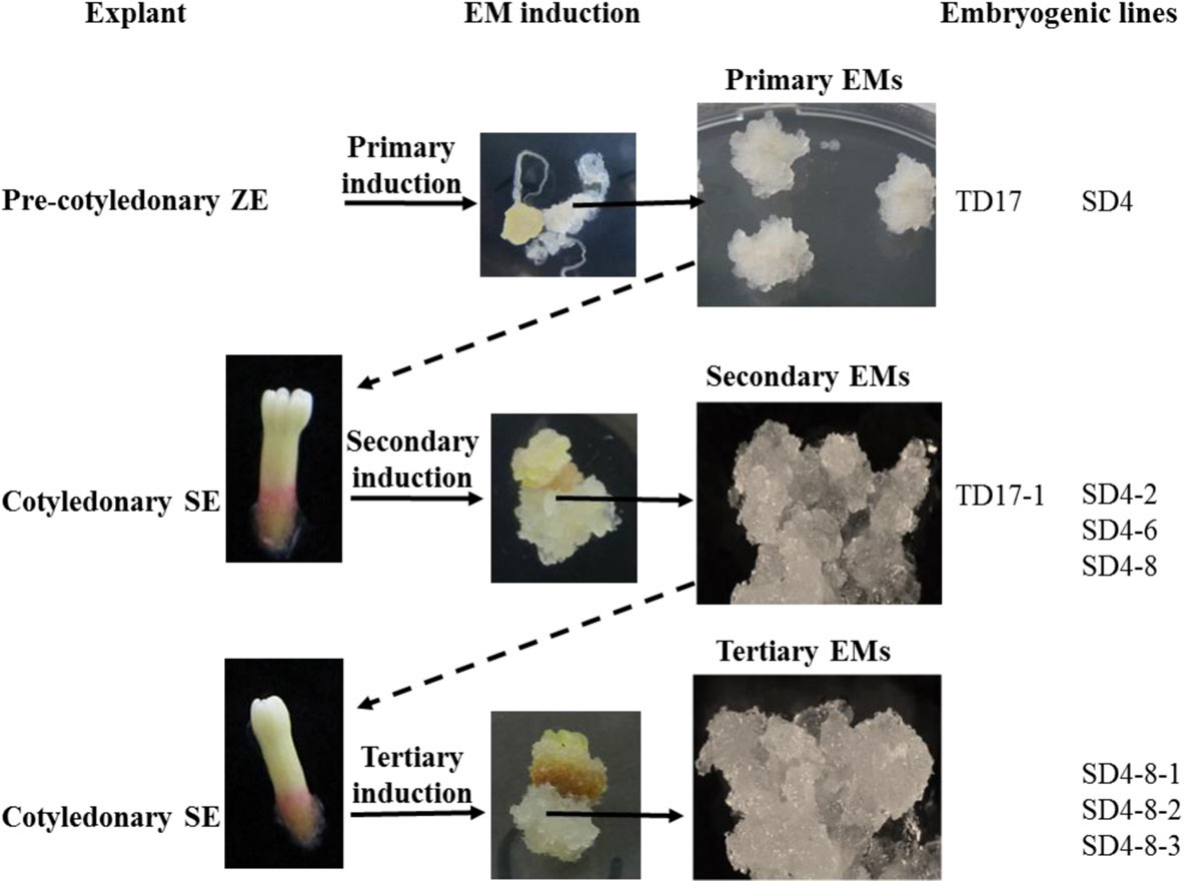
Origin of the Douglas-fir material analyzed in this work (1^ry^, 2^ry^ and 3^ry^ embryogenic lines). EMs: embryonal masses; SE: somatic embryo; ZE: zygotic embryo

#### Morphological and histo-cytological observations during proliferation

Samples were collected after 10 days of multiplication for morphological and histo-cytological characterizations. Morphology of EMs was documented using a SMZ 1500 stereomicroscope (Nikon, Tokyo, Japan), and their structure was examined using a Jenaval transmission light microscope (Zeiss, Jena, Germany) after staining fresh material with 0.4% (*w*/*v*) Trypan Blue (Sigma-Aldrich), as previously described [47]. Starch grains were localized by staining with Lugol (iodine-potassium iodide) solution. Paraffin sections (12 μm thick) of EMs samples stained with Alcian Blue and Nuclear Fast Red following fixation, dehydration and paraffin infiltration [6], were observed under a Jenaval light microscope for histological observations. In addition to cell walls, Alcian Blue may stain vacuolar contents of some cells, and EMs’ colors indicate that some may accumulate phenolic compounds. Therefore, Azur II and Safranin dyes (known to stain phenolic compounds) were also used, and confirmed that some of the Alcian Blue staining was due to its complexation with phenolics. All images were captured using a DS-5 M camera (Nikon, Tokyo, Japan) and processed using NIS-Elements AR 3.2 image analysis system (Laboratory Imaging, Prague, Czech Republic).

#### Maturation conditions

Proliferating EMs from filter papers were weighed, dispersed in liquid Glitz medium without plant growth regulators and distributed on a filter paper disc placed on the surface of Glitz maturation medium (in a Petri dish) supplemented with 0.2 M sucrose, 60 μM ABA (cis-trans ± abscisic acid), and 10 g L^− 1^ gellan gum at a cell density of 50 mg f.w. per filter. The EMs were then matured in darkness at approximatively 23 °C. The number of cotyledonary SEs generated in each Petri dish after 8 weeks was counted, and EMs’ embryogenic potential (number of SEs per g f.w. EMs) was estimated. In a first set of experiments, the embryogenic potential of 1^ry^ lines (SD4, TD17) and 2^ry^ lines (SD4–2, SD4–6, SD4–8, TD17–1) were compared using 96 Petri dishes (5–6 for each permutation of line and conditions, with sets of three biological replicates). In a second set of experiments we compared the embryogenic potential of 2^ry^ (SD4–8) and 3^ry^ (SD4–8-1, SD4–8-2, SD-4-8-3) lines, using 6 Petri dishes for each permutation of lines and conditions, and experiments were repeated three times, so 72 Petri dishes were used in total.

#### Soluble proteins extraction

Analyses were performed for all types of lines (1^ry^, 2^ry^ and 3^ry^), with EMs cultured as a thin layer dispersed onto filter paper. Soluble proteins extracts were prepared from four biological replicates for each samples (150 mg f.w. of frozen EMs) with 1 ml of urea extraction buffer (4 M urea, 0.1% v/v SDS 10%, 0.1 M DTT, 80 mM Tris HCl pH 6.8, 10% v/v glycerol). Total protein content was determined using the Bradford assay with bovine serum albumin as a standard. Results were expressed as soluble proteins content (μg mg^− 1^ of dry weight).

#### Proteomic and label-free quantitative MS/MS data analyses

Proteomic and nLC-MS/MS analyses were performed following previously published protocols [48]. Briefly, protein samples were subjected to SDS-PAGE and stained with colloidal blue, then stained bands were cut from the gel, destained and digested by trypsin. The resulting peptide mixtures were analyzed using an Ultimate 3000 nanoLC system (C18 PepMapTM trap column, Dionex, Amsterdam, The Netherlands) coupled to an Electrospray Q-Exactive quadrupole Orbitrap benchtop mass spectrometer (Thermo Fisher Scientific, San Jose, CA). The mass spectrometer was operated in positive ion mode at 1.8 kV needle voltage. Data were acquired using Xcalibur 2.2 software in a data-dependent mode. MS scans (m/z 350–1600) were recorded at a resolution of *R* = 70,000 (at m/z 200) with an AGC target of 3E6 ions collected within 100 ms. Dynamic exclusion was set to 30 s and the top 15 ions were selected from fragmentation in HCD mode. MS/MS scans with a target value of 1E5 ions were collected with a maximum fill time of 100 ms and resolution of *R* = 17,500. Additionally, only + 2 and + 3 charged ions were selected for fragmentation. Acquired peptide were searched by SEQUEST implemented in Proteome Discoverer 1.4 (Thermo Fisher Scientific Inc.) against a *Pseudotsuga menziesii* v1 transcriptome – proteome database from PineRefSeq (54,595 entries, August 2016, https://treegenesdb.org/FTP/Genomes/Psme/v1.0/annotation/). Spectra from peptides larger than 5000 Da or smaller than 350 Da were rejected and mass accuracy of the monoisotopic peptide precursor and peptide fragments was set to 10 ppm and 0.02 Da, respectively. Only b- and y-ions were considered for mass calculations. Oxidation of methionine (+ 16 Da) was considered as a variable modification and carbamidomethylation of cysteines (+ 57 Da) as a fixed modification. Two missed trypsin cleavages were allowed. Peptide were using the Percolator algorithm [49] and only “high confidence” peptides were retained, corresponding to a < 1% False Discovery Rate (FDR) at peptide level. Raw LC-MS/MS data were imported into Progenesis QI for Proteomics 2.0 (Nonlinear Dynamics Ltd., Newcastle, U.K). Data processing included the following steps: (i) features detection, (ii) features alignment across all samples, (iii) volume integration for 2–6 charge-state ions, (iv) raw data normalization based on medians of ratios of all intensities of relevant fragments to references (calculated from LC-MS features), (v) import of sequence information, and calculation of protein abundance (sums of volumes of corresponding peptides). Only non-conflicting features and unique peptides were considered for protein level calculation. Quantitative data were considered for proteins quantified by a minimum of two peptides. The mass spectrometry proteomics data have been deposited in the ProteomeXchange Consortium databasevia the PRIDE [50] partner repository with the dataset identifier PXD008347.

#### Functional characterization and gene ontology analysis

Changes in expression, relative to appropriate controls, were calculated based on the cumulative intensity of each peptide (classifying proteins with ≥1.5-fold change ratios as up- or down-regulated). All sequences were mapped against Gene Ontology (GO) terms in the TAIR *Arabidopsis thaliana* database (https://www.arabidopsis.org/) for functional annotation. The proteins were then classified based on their biological functions using Web Gene Ontology Annotation Plot software at level 2 for biological processes (Panther, http://pantherdb.org/) [51]. A binomial test and Bonferroni’s correction were applied with Panther software to identify classes represented significantly more frequently than expected among up- and down-regulated proteins in each type of material. As the gene ontology is currently extremely poor for Douglas-fir, we also applied another method to assess enrichment of GO terms in our protein sets, using the Bioconductor R package topGO 2.26.0 [52], based on the “weight” method and Fisher’s exact test. We compared the sets of significant proteins against the 4813 Douglas protein total dataset, and mapped each protein to the best Arabidopsis homolog by BlastP searches. Then, each GO term from *Arabidopsis thaliana* was associated with the corresponding Douglas protein for topGO analysis.

#### Network enrichment analysis

Sub-Network Enrichment Analysis (SNEA) was performed using Pathway Studio® version 11.4 (Elsevier B.V.).

#### Statistical analyses

R software (version 3.3.2; R Development Core team 2011) was used for all statistical analyzes. Embryogenic potential and the soluble protein contents of 1^ry^, 2^ry^ and 3^ry^ lines were evaluated using one-way analysis of variance (ANOVA) and multiple comparisons of means with Tukey contrasts (*P* < 0.05). For proteomic analysis, differential expression of proteins in 1^ry^ vs 2^ry^ lines, and 2^ry^ vs 3^ry^ lines, was analyzed using two-way ANOVA with interaction and FDR, based on normalized abundance (adjusted *P* < 0.05).

## Results

Embryogenic potential of Douglas-fir embryogenic lines after repetitive somatic embryogenesis.

### Secondary vs primary embryogenic lines of TD17 and SD4 genotypes

In the initial comparison of the embryogenic potential of 1^ry^ (TD17 and SD4) and 2^ry^ (TD17–1, SD4–2, SD4–6 and SD4–8) lines, the primary lines showed significant variations in mean production of cotyledonary SEs (*P* = 3.01e^− 7^). SD4 was moderately embryogenic (478 SEs g^− 1^ f.w.) whereas TD17 showed very weak embryogenic potential (30 SEs g^− 1^ f.w., Table 1). However, in both cases 2^ry^ lines were significantly more productive than 1^ry^ lines (SD4 *P* = 9.47e^− 7^ and TD17 *P* = 2.88e^− 10^). TD17–1 was 8 times more productive (generating 241 SEs g^− 1^ f.w., Table 1) than the original TD17 line, while 2^ry^ lines of SD4 yielded 3–4 (SD4–6, SD4–8) to 6 times (SD4–2) more cotyledonary SEs (1515–3131 SEs g^− 1^ f.w. Table 1).

**Table 1:** Mean yield in cotyledonary somatic embryos (SEs) of Douglas-fir from 1^ry^ and 2^ry^ embryogenic lines

Line	Mean no. of SEs g^− 1^ f.w.	
Primary		
TD17	30	± 19^a^
SD4	478	± 139^c^
Secondary		
TD17–1	241	± 70^b^
SD4–2	3131	± 34^e^
SD4–6	1515	± 297^d^
SD4–8	1821	± 363^d^

Values are means of 3 biological and 5–6 technical replicates ±95% confidence limits. Significant differences (*p* < 0.05) in multiple comparisons of means are indicated by different letters

### Tertiary vs secondary embryogenic lines of SD4 genotype

In the subsequent comparison of the embryogenic potential of 2^ry^ (SD4–8) and 3^ry^ (SD4–8-1, SD4–8-2 and SD4–8-3) lines, SE yields of the three 3^ry^ lines were very high (3344–4258 SEs g^− 1^ f.w., Table 2). However, only SD4–8-2 and SD4–8-3 had significantly higher (*P* = 0.00337) yields (4160 and 4258 SEs g^− 1^ f.w., respectively) than SD4–8 (2401 SEs g^− 1^ f.w.).

**Table 2:** Mean yield in cotyledonary somatic embryos (SEs) of Douglas-fir from 2^ry^ and 3^ry^ embryogenic lines

Line	Mean no. of SEs g^−1^ f.w.	
Secondary		
SD4–8	2401	± 534^a^
Tertiary		
SD4–8-1	3344	± 1274^ab^
SD4–8-2	4160	± 931^b^
SD4–8-3	4258	± 829^b^

Values are means of 3 biological and 5–6 technical replicates ±95% confidence limits. Significant differences (*p* < 0.05) in multiple comparisons of means are indicated by different letters

#### Histo–cytological description of Douglas-fir EMs

Macromorphological (EM color and morphotype) and histo-cytological traits (occurrence of polyembryogenic centers, singulated SEs and NECs) of primary (SD4, TD17), secondary (SD4–2, 6, 8; TD17–1) and tertiary (SD4–8-1, 2, 3) embryogenic lines of Douglas-fir are summarized in Additional file 1: Table S1.

### Morphology of primary, secondary and tertiary EMs

EMs of both genotypes and all types of lines (1^ry^, 2^ry^, 3^ry^) had various colors; usually shades of yellow and brown, but some lines (especially SD4) were rather pink (Additional file 2: Figure S1) indicating local accumulation of phenolic compounds in their cells, as confirmed by histological-level histochemical staining (see below), and activation of phenolic pathways in the 1^ry^ lines (see below). The structure of immature early SEs proliferating in EMs was hardly distinguishable in the 1^ry^ lines. However, the EMs’ surfaces had notable granularity, particularly those of line SD4 (Additional file 2: Figure S1), which could indicate occurrence of large polyembryogenic centers. Detailed observations also revealed filamentous suspensor cells attached to a few of these structures escaping from the EMs’ surfaces. In contrast, distinct, compact and granular large polyembryogenic centers and/or whitish singulated early SEs were apparent on most EMs of 2^ry^ and 3^ry^ lines, especially those of lines TD17–1, SD4–2, SD4–8-1 and SD4–8-3, in which the bipolar structure of protruding early embryos could sometimes be distinguished (Additional file 2: Figure S1, arrows). EMs from 2^ry^ line SD4–6 resembled those of the 1^ry^ line, while EMs of SD4–8 did not exhibit a typical granular morphotype and had a smoother appearance, suggesting that polyembryogenic centers or singulated embryos of this line are less frequent and/or smaller (Additional file 2: Figure S1). EMs from 3^ry^ line SD4–8-2 most resembled EMs from 2^ry^ line SD4–2, but had fewer distinct structures with granular appearance.

#### Histological comparison of secondary (TD17–1, SD4–2, SD4–6, SD4–8) and primary (SD4, TD17) embryogenic lines

**Fig. 2:**
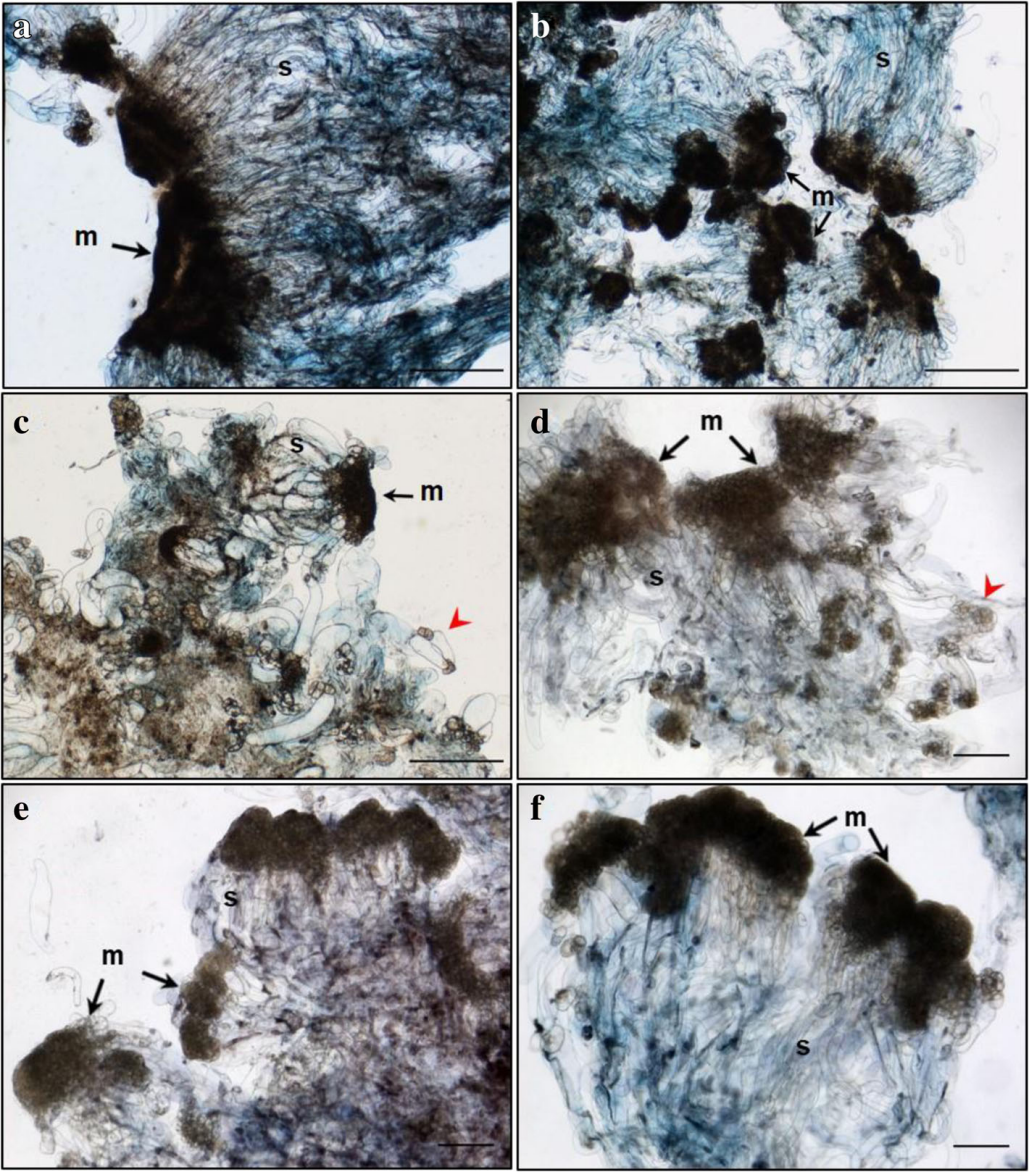
Structure of embryonal masses from primary, secondary and tertiary lines of genotype SD4. **a** / SD4; **b** / SD4–2; **c** / SD4–8; **d** / SD4–8-1; **e** / SD4–8-2 **f** / SD4–8-3. Arrowheads in **c**, **d** mark small singulated somatic embryos; m – meristem of polyembryogenic centers, s – suspensor; Trypan blue staining of squashes of fresh EMs. Scale bar = 500 μm

**Fig. 3:**
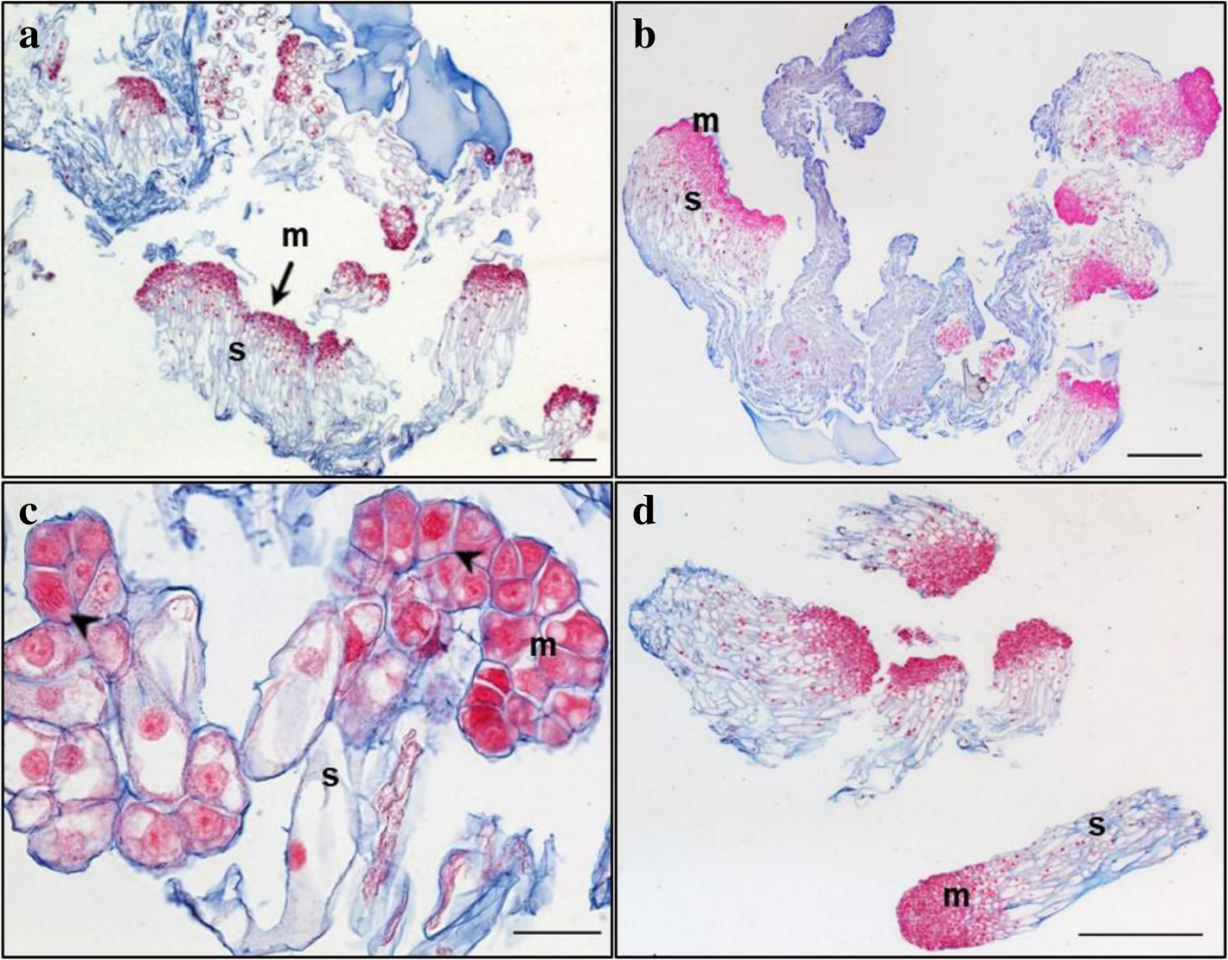
Histology of embryonal masses from primary and secondary lines of the genotype TD17. **a**, **c** / TD17; **b**, **d** / TD17–1. **a**,**b** – polyembryogenic centers (PECs); **c** – small SEs, arrowheads point to actively dividing cells (metaphase/anaphase); **d** – singulated large SEs; m – meristem of PECs or singulated SEs, s – suspensor; Paraffin sections stained with Alcian Blue/Nuclear Fast Red. Scale bars: **a**, **d**, **f** = 500 μm; **b** = 100 μm; **c** = 200 μm; **e** = 50 μm

**Fig. 4:**
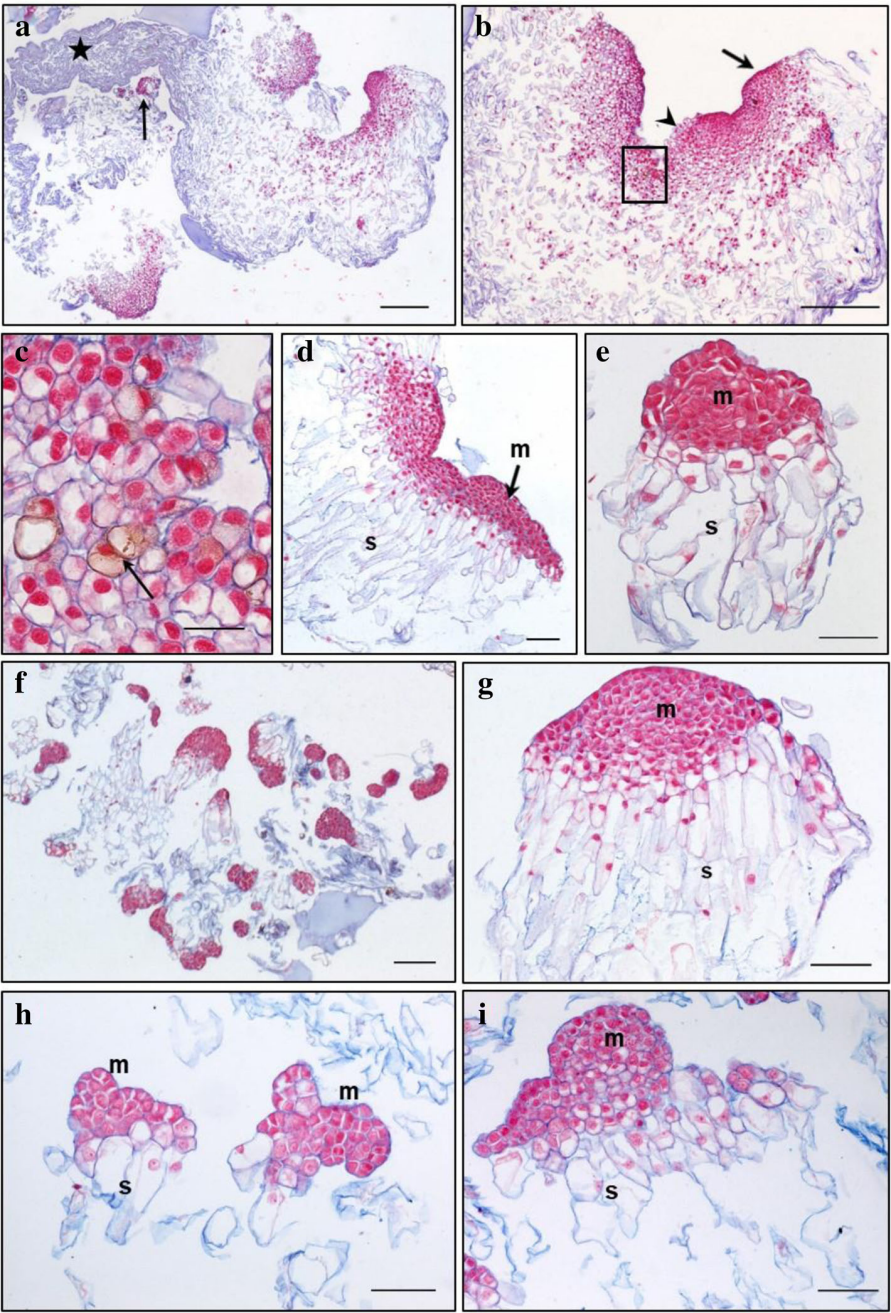
Histology of embryonal masses from primary and secondary lines of the genotype SD4. **a**, **b**, **c** / SD4; **d**, **e** / SD4–2; **f**, **g** / SD4–6; **h**, **i** / SD4–8. **a**, **b** – structures resembling the polyembryogenic centers (PECs) Arrow in **a** points to small somatic embryos (SEs) and the star marks the dead material in the end of suspensor region; arrow in **b** points to the smooth surface of protoderm and arrowhead marks the place where protoderm is missing; the detail of the framed region in **b** is shown in **c**; arrow in **c** points to the brown cells with phenolic content located in the meristem-like region; **d** – PEC; **e** – singulated SEs, **f** – cluster of small SEs and PECs; **g** – well-organized SEs; **h** – small SEs; **i** – small PECs; m – meristem of PECs or singulated SEs, s – suspensor. Paraffin sections stained with Alcian Blue/Nuclear Fast Red Scale bars: **a**, **b** = 500 μm; **c** = 50 μm; **d**, **e**, **g**, **h**, *I* = 100 μm; **f** = 200 μm

**Fig. 5:**
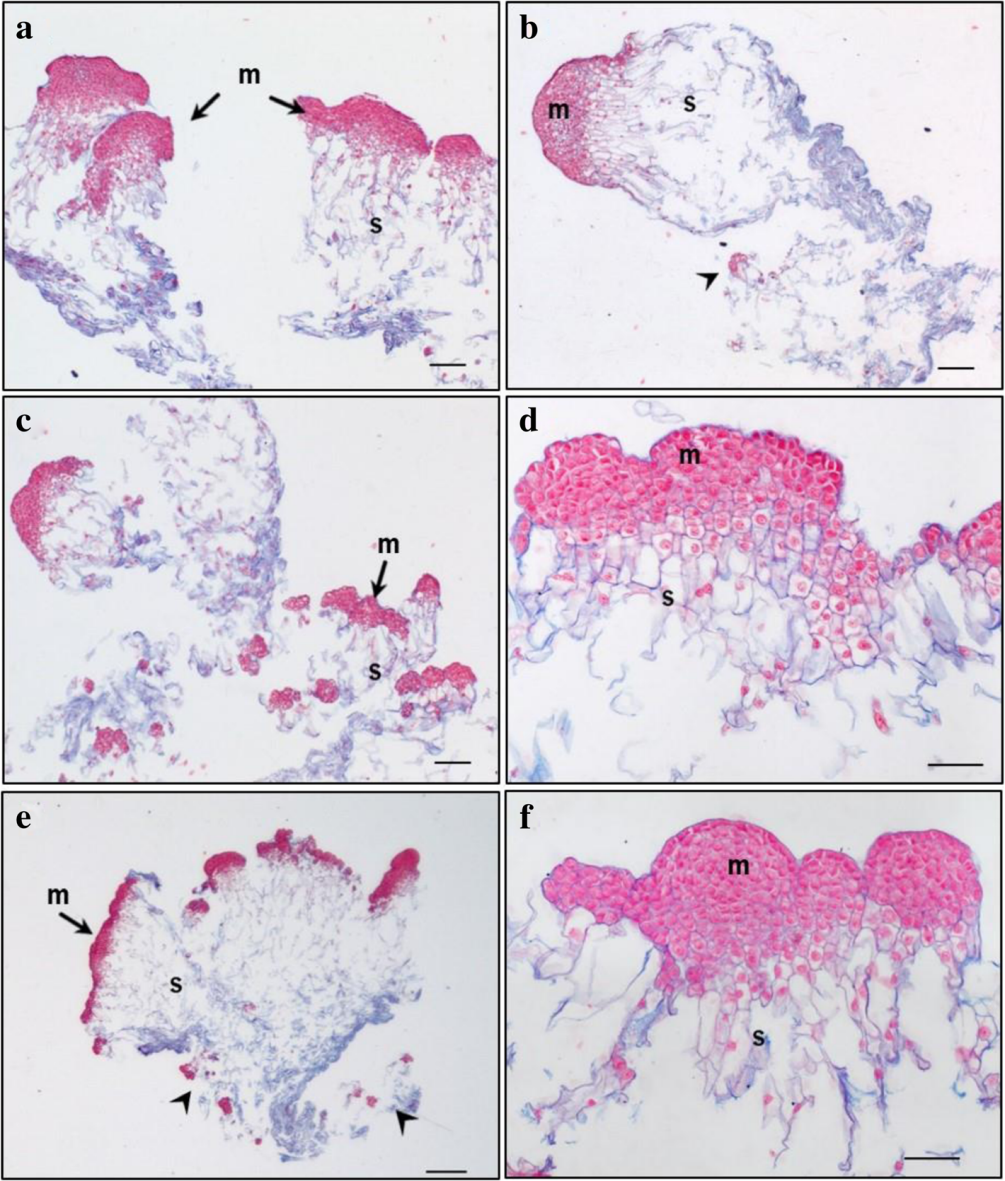
Histology of embryonal masses from tertiary lines of the genotype SD4. **a**, **b** / SD4–8-1; **c**,**d** / SD4–8-2; **e**,**f** / SD4–8-3. **a**, **b** – Polyembryogenic centers (PECs) Arrowheads in **b** points to small SEs; **c** – PECs, smaller ones with distinct embryonal heads; **d** – detail of PEC; **e** – PECs, arrowheads points to small SEs; **f** – detail of PEC with well-organized embryonal heads; m – meristem of PEC, s – suspensor. Paraffin sections stained with Alcian Blue/Nuclear Fast Red. Scale bar: **a**, **b**, **c** = 200 μm; **d**, F = 100 μm; **e** = 500 μm

Primary lines of both genotypes TD17 and SD4 produced large polyembryogenic centers, with broad meristematic parts and usually elongated suspensor cells, collectively creating compact cell “packages” with cells joined together by a mucilaginous matrix (Figs. 2, 3, 4 and 5), Additional file 3: Figure S2). Besides these polyembryogenic centers, singulated early embryos with a large well-organized embryonal head connected to a compact and long suspensor were also formed (Fig. 3d). Smaller embryos composed of several layers of meristematic cells and a few elongated suspensor cells were also often found in, or close to, dead material formed by remnants of suspensor cells or disintegrated early embryos (Figs. 2c, d, 4a, h, 5b, e). Meristematic cells of these small embryos were usually mitotically active (Fig. 3c).

Both genotypes also produced clusters of NECs, as either groups of loosely arranged vacuolated cells (Additional file 4: Figure S3A) or organized, compact cell aggregates (Additional file 4: Figure S3B), both located in the proximity of early embryos. All observed cell clusters were examined very carefully in successive sections to avoid possible confusion of NECs with embryonal cells, especially with suspensor cells, which are also vacuolated. Cell clusters were considered non-embryogenic if none of their cells in any section exhibited characteristics of meristematic cells, i.e. dense cytoplasm, prominent nuclei and small vacuoles with no detectable phenolic content. Large NEC clusters usually consisted of a mixture of irregularly shaped vacuolated cells that were either mitotically active (Additional file 4: Figure S3) or accumulated starch grains and/or phenolic compounds (Additional file 4: Figure S3B,C,E,F). Phenolic compounds were apparently deposited in vacuoles as granules or droplets, resulting in brownish cells that were easily recognizable in aggregates with cell walls stained blue and both nuclei and cytoplasm (slightly) stained pink/red (see Fig. 4c). Surprisingly, phenolics accumulating in vacuoles as amorphous deposits reacted differentially with Alcian Blue, forming complexes of various colors from grey-blue to ginger, suggesting they had variable chemical composition (Additional file 4: Figure S3B,E,F). Alcian Blue staining of phenolic compounds was confirmed histochemically by other dyes (Azur II and Safranin; data not shown). The occurrence of NEC aggregates interspersed in EMs was found to vary between lines as described below. However, repetitive somatic embryogenesis globally resulted in reduced frequency of NEC clusters and, concurrently, in reduced phenolic contents in EMs.

#### TD17 vs TD17–1

Both 1^ry^ and 2^ry^ lines of genotype TD17 produced all types of embryogenic structures mentioned above (Additional file 3: Figure S2, Fig. 3), but most frequently large polyembryogenic centers (Fig. 3a, b), while small embryos (Fig. 3c) were quite rare in both lines. Large singulated SEs with a well-organized embryonal head were more frequent in the 2^ry^ line TD17–1 (Fig. 3d). NECs were observed in TD17 as loosely arranged cells (Additional file 4: Figure S3A) or compact cell clusters (Additional file 4: Figure S3B) in the vicinity of embryos or even in the dead material. The TD17–1 2^ry^ line produced much fewer NEC clusters. Only small pieces were observed close to early embryos or (more often) in the dead material.

#### SD4 vs SD4–2, SD4–6, SD4–8

Primary line SD4 is characterized by the production of large structures resembling polyembryogenic centers (Figs. 2a, 4a, b). The meristem-like parts of these structures were usually formed by a mixture of densely cytoplasmic meristematic cells and vacuolated cells that often accumulated starch grains and/or phenolic compounds (Fig. 4c). Meristematic cells usually occurred in one part of the structure forming a compact meristemoid or embryonal head-like structure. This was probably only a small part or a few cell layers of the polyembryogenic centers, as the inner region was mainly formed by vacuolated cells. Protodermal cells usually created smooth surfaces of this meristem-like region (Fig. 4b). However, the outermost cell layer could deteriorate in some parts, resulting in missing protoderm and local cell organization very similar to that of NEC clusters (Fig. 4b). Elongated suspensor cells were only present in the outermost region of the cell “package” or simply missing. The inner region, located distally from the meristem-like region, consisted of loosely arranged, irregularly shaped cells. Small embryos with a typical bipolar arrangement of meristematic and suspensor cells occurred rarely among the dead remnants of suspensor cells in the most distal region of the structure (Fig. 4a).

In contrast to the 1^ry^ line, 2^ry^ lines produced well-arranged polyembryogenic centers of various sizes formed by compact meristematic parts joined together with elongated suspensor cells. The most typical polyembryogenic centers with distinct embryonal heads were observed in SD4–2 (Figs. 2b, 4d) together with small individual embryos located in suspensors (Fig. 4e). In this line, additional large structures arranged in a similar way to polyembryogenic centers were observed, but some parts were composed of vacuolated cells with starch grains resembling NECs. On the edges of these structures, cells accumulated phenolic compounds creating a frontier between a compact meristem-like part and suspensor cells (Additional file 4: Figure S3E). SD4–6 material consists in numerous small embryos or small polyembryogenic centers with noticeable embryonal heads (Fig. 4f,g) as well as a few polyembryogenic centers of huge size. There were numerous large compact pieces of NECs located in the vicinity of embryos. Large, very compact and highly organized cell structures resembling meristemoids were the most typical types of non-embryogenic structure produced by line SD4–6 (Additional file 4: Figure S3F). These “meristemoids” were isolated from other parts of the clumps by groups of cells with high phenolic contents. Line SD4–8 only produced small embryos and smaller polyembryogenic centers that were not very well organized (Fig. 4h,i). Like other 2^ry^ lines, SD4–8 also produced NECs, arranged in clusters of vacuolated cells accumulating starch grains and/or phenolics, similar to those observed in TD17 (Additional file 4: Figure S3B).

#### Histological comparison of tertiary (SD4–8-1; SD4–8-2; SD4–8-3) and secondary (SD4–8) embryogenic lines

Tertiary lines produced more, and much bigger, polyembryogenic centers but fewer NEC clusters than the 2^ry^ line SD4–8. Line SD4–8-1 produced large polyembryogenic centers and clusters of large singulated early embryos with distinct embryonal heads (Figs. 2d, 5a,b) and small embryos within suspensors (Fig. 5b). Lines SD4–8-2 typically produced large numbers of smaller polyembryogenic centers and smaller embryos (Fig. 2e, 5c,d) while large polyembryogenic centers were found less organized with some signs of disintegration of both meristems and suspensors. Line SD4–8-3 was characterized by large polyembryogenic centers with numerous distinct embryonal heads joined to very dense suspensors formed of elongated cells. Each of these polyembryogenic centers was attached to another by an “anchor” of dead suspensor cells. Small embryos appeared within or in the vicinity of suspensors (Figs. 2c, 5e,f).

### Total protein content and proteomic analysis

#### Total protein content

The total protein content was similar in both genotypes SD4 and TD17 (mean values: 113.4 and 98.3 μg mg^− 1^ d.w., respectively) and in both 1^ry^ and 2^ry^ lines (mean values: 107.2 and 108.9 μg mg^− 1^ d.w., respectively, Additional file 5: Table S2). Higher protein contents were detected in 3^ry^ than in 2^ry^ lines (mean values: 137.5 and 110.8 μg mg^− 1^ d.w., respectively, Additional file 6: Table S3) of genotype SD4, but the observed difference is not significant.

#### Proteomic comparisons of secondary (TD17–1; SD4–2; SD4–6; SD4–8) vs primary (SD4, TD17) and tertiary (SD4–8-1; SD4–8-2; SD4–8-3) vs secondary (SD4–8) embryogenic lines

**Fig. 6:**
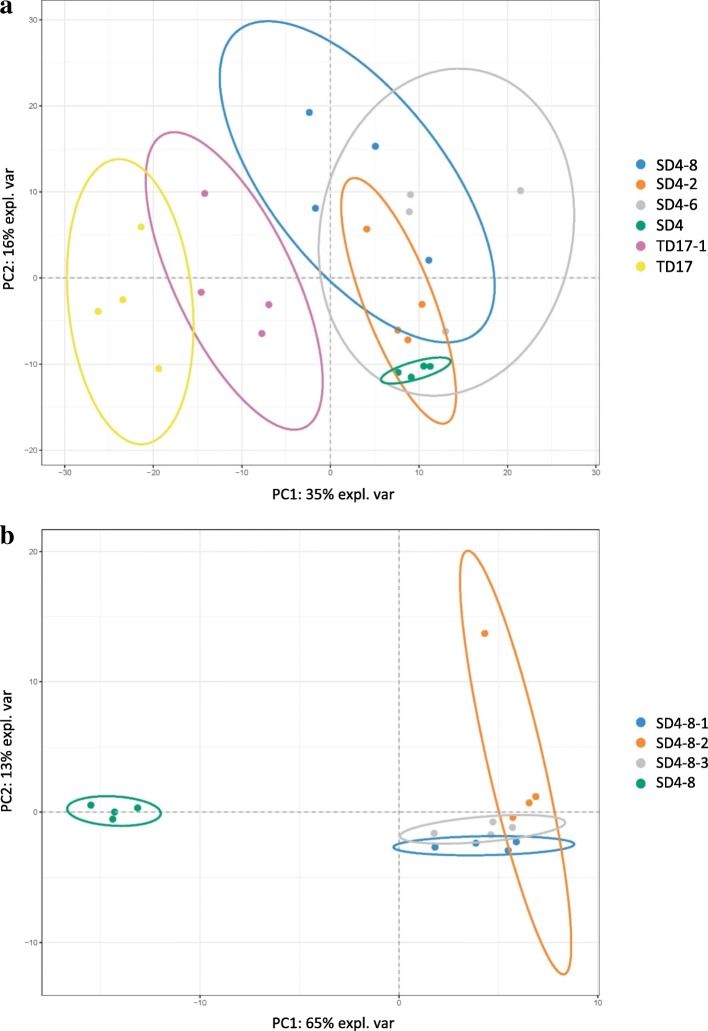
Principal component analysis of proteins identified during the proteomic studies. **a** / Comparison between the 1^ry^ and 2^ry^ lines; **b** / Comparison between 2^ry^ (SD4–8) and 3^ry^ lines (SD4–8-1, SD4–8-2, SD4–8-3)

In the LC-MS/MS analysis of total protein extracts and changes in protein expression (detailed in the Methods section) we identified 2293 proteins in the comparison of 2^ry^ vs 1^ry^ lines, and 2554 in the comparison of 3^ry^ vs 2^ry^ lines, so a set of 4813 unique proteins. Principal component analysis (PCA) is a multivariate technique that analyzes a data set with inter-correlated quantitative dependent variables to represent it as a set of new orthogonal variables called principal components displaying the pattern of similarities. The first principal component (PC1 axis) obtained from PCA of the 2293 proteins identified in 1^ry^ and 2^ry^ lines (Fig. 6a) was mainly related to the genotype and explained 35% of the total variance. PC2 axis was mainly related to interaction between genotype and type of line (1^ry^, 2^ry^), and explained 16% of the total variance. PC1 obtained from PCA of all 2554 proteins identified in 2^ry^ and 3^ry^ lines of genotype SD4 explained 65% of the total observed variance and type of line was the main determinant (Fig. 6b).

In the subsequent two-way ANOVA of changes in protein expression following the first and second cycles of somatic embryogenesis, 162 and 288 significant proteins were identified in the comparisons of 2^ry^ vs 1^ry^ lines and 3^ry^ vs 2^ry^ lines, respectively (Additional file 7). Most significant proteins were down-regulated in 2^ry^ relative to 1^ry^ lines, but up-regulated in 3^ry^ relative to 2^ry^ lines (76 and 70% of the sets, respectively). Surprisingly, only 33 proteins were members of both sets, moreover 9 of these 33 were up-regulated following both somatic embryogenesis cycles, 3 were down-regulated following both cycles and there were opposite changes in expression of the other 21 proteins. Thus, specific sets of differentially expressed proteins, and changes in expression profiles, were associated with each cycle of somatic embryogenesis. In Additional file 8: Table S4 presents the functional GO classifications of the 162 (2^ry^ vs 1^ry^) and 228 (3^ry^ vs 2^ry^) significant proteins. The most strongly represented categories are “Metabolic Processes”, “Cellular Processes” and “Response to Stimulus” (respectively accounting for 52.9, 27.3 and 6.6% of the first set, and 46.0, 30.7 and 7.4% of the second set).

**Fig. 7:**
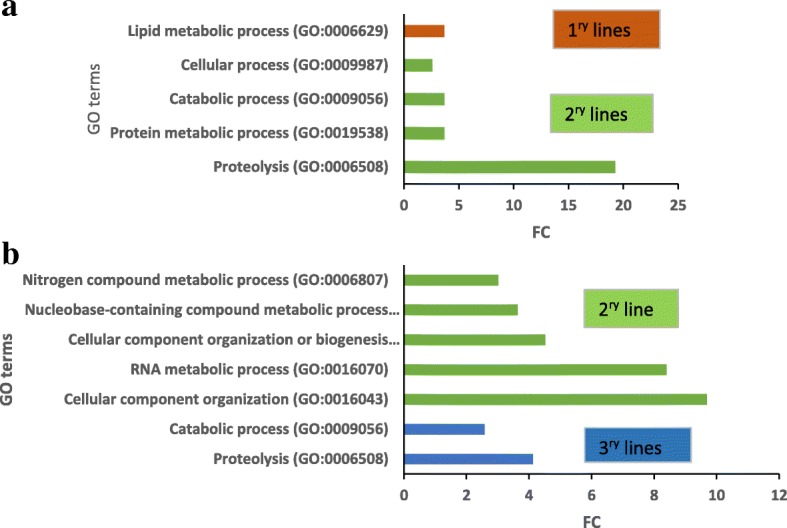
Enrichment analysis of GO (Gene ontology) terms of the Biological Process categories (at level 2 or more). GO terms found to be significantly enriched between **a**/ 1^ry^ (TD17, SD4) and 2^ry^ (TD17–1, SD4–2, SD4–6, SD4–8) embryogenic lines or **b**/ 2^ry^ (SD4–8) and 3^ry^ (SD4–8-1, SD4–8-2, SD4–8-3) embryogenic lines of Douglas-fir. Fold change (FC) corresponds to the ratio of the number of identified hits between 2^ry^ and 1^ry^ or 3^ry^ and 2^ry^ lines, respectively

**Fig. 8:**
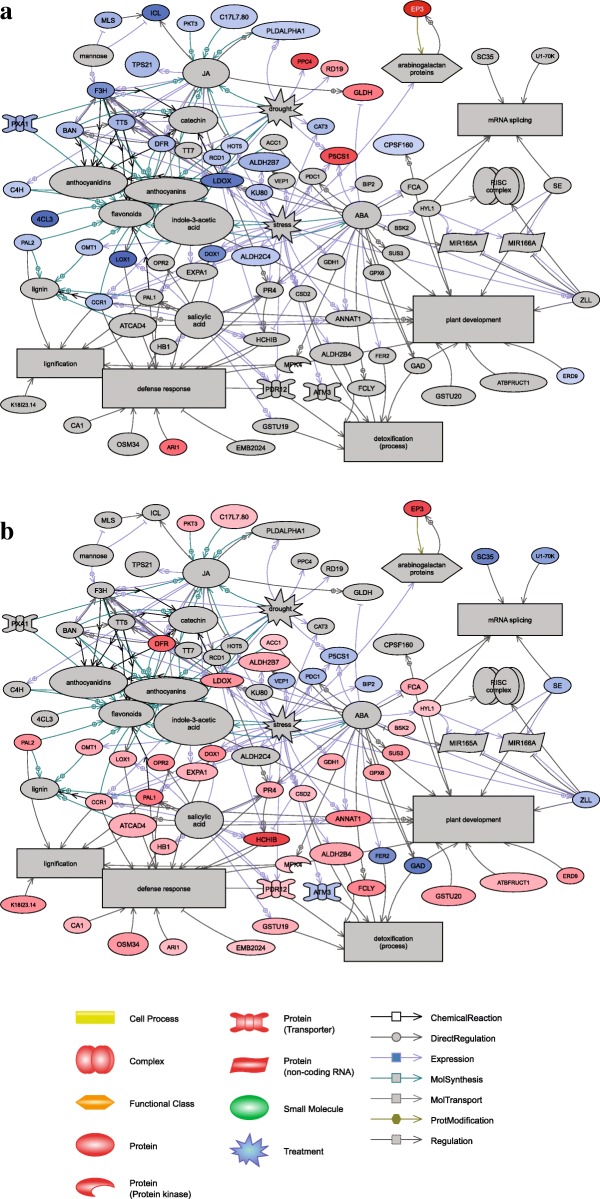
Sub Network Enrichment Analysis (SNEA) connecting significant proteins and regulators or targets from proteomic studies. **a** / Comparison of 2^ry^ vs 1^ry^ lines; **b** / Comparison of 3^ry^ vs 2^ry^ lines somatic embryogenesis in Douglas-fir. The Douglas-fir significant proteins (ratio 1.5, *p* < 0.05) are named by their *Arabidopsis* homologues. The correspondences between Douglas-fir and *Arabidopsis* protein names are given in Additional file 1. Red color: sur-expression of this protein in 2^ry^ lines in **a** and 3^ry^ lines in **b**; blue color: sur-expression of this protein in 1^ry^ lines in **a**, 2^ry^ line in **b**; grey color: protein significant in the proteomic comparison

Results of GO category enrichment analyses (Term Enrichment) using Panther and Biocoductor R of overall trends of the functional categories enriched in the lines following the cycles are shown in Table 3 and Fig. 7, respectively. Proteins involved in “Proteolysis” and “Catabolic process” categories were over-represented in both sets of significant proteins (Fig. 7). Many GO terms are enriched in significant proteins associated with both cycles of somatic embryogenesis (Table 3), including processes involved in chitin and polysaccharide catabolism, lignin and L-phenylalanine metabolism, phenylpropanoid biosynthesis, oxidation-reduction, and response to karrikin. Strikingly, the expression of proteins assigned to these GO term categories declined following the first cycle but increased after the second cycle, corroborating the finding that different protein profiles were established after each cycle of somatic embryogenesis. The SNEA of significant proteins is based on *Arabidopsis thaliana* bibliographic database, which implies the network representation with *Arabidopsis* protein names. The SNEA provided further indications of their functions, interactions and putative targets, as well as regulators involved in metabolic pathways that may be affected during repetitive somatic embryogenesis (Fig. 8a,b). Overall, 70 significant proteins up- or down-regulated after 2^ry^ (33 proteins) or 3^ry^ somatic embryogenesis (51 proteins) were integrated in the constructed networks. They included many proteins associated with metabolism of plant growth signaling compounds, including plant growth regulators such as jasmonic acid, ABA and salicylic acid, together with proteins related to lignin (especially after 3^ry^ somatic embryogenesis) and flavonoid secondary metabolites (especially after 2^ry^ somatic embryogenesis). Indole-3-acetic acid (IAA) was revealed as an important regulator associated with both networks. The subsets of significant proteins and targets/regulators in each network were mostly specific for each cycle of somatic embryogenesis, but affected the same major biological processes (“plant development” and “defense response”). In the latter case, plant growth signaling compounds, such as jasmonic acid, salicylic acid and ABA and many other molecules identified in these networks (including secondary metabolites) have known involvement not only in adaptation to stress (defense responses, detoxification and drought stress/hyperosmotic salinity responses), but also in non-defensive functions during plant development.

**Table 3:** Gene Ontology (GO) terms enriched in significant proteins after repetitive somatic embryogenesis in Douglas-fir

GO.ID	Term	Annot.	Sign.	Exp.	ratio sign./exp.	Expression
Secondary versus primary lines						
GO:0015977	carbon fixation	17	2	0.13	15.38	2^ry^ > 1^ry^
GO:0009269	response to desiccation	17	2	0.13	15.38	2^ry^ > 1^ry^
GO:0006099	tricarboxylic acid cycle	38	2	0.28	7.14	2^ry^ > 1^ry^
GO:0015979	photosynthesis	43	2	0.32	6.25	2^ry^ > 1^ry^
GO:0051603	proteolysis involved in cellular protein catabolic process	142	5	1.05	4.76	2^ry^ > 1^ry^
GO:0043488	regulation of mRNA stability	2	2	0.05	40.00	1^ry^ > 2^ry^
GO:0046292	formaldehyde metabolic process	4	2	0.1	20.00	1^ry^ > 2^ry^
GO:0006556	S-adenosylmethionine biosynthetic process	7	3	0.18	16.67	1^ry^ > 2^ry^
GO:0006097	glyoxylate cycle	7	3	0.18	16.67	1^ry^ > 2^ry^
GO:0010030	positive regulation of seed germination	5	2	0.13	15.38	1^ry^ > 2^ry^
GO:0051262	protein tetramerization	6	2	0.15	13.33	1^ry^ > 2^ry^
GO:1901663	quinone biosynthetic process	13	4	0.33	12.12	1^ry^ > 2^ry^
GO:0006558	L-phenylalanine metabolic process	15	4	0.38	10.53	1^ry^ > 2^ry^
GO:0009969	xyloglucan biosynthetic process	8	2	0.2	10.00	1^ry^ > 2^ry^
GO:0009051	pentose-phosphate shunt, oxidative branch	8	2	0.2	10.00	1^ry^ > 2^ry^
GO:0042593	glucose homeostasis	8	2	0.2	10.00	1^ry^ > 2^ry^
GO:0010262	somatic embryogenesis	14	3	0.35	8.57	1^ry^ > 2^ry^
GO:0006032	chitin catabolic process	22	4	0.55	7.27	1^ry^ > 2^ry^
GO:0009813	flavonoid biosynthetic process	58	9	1.46	6.16	1^ry^ > 2^ry^
GO:0002215	defense response to nematode	13	2	0.33	6.06	1^ry^ > 2^ry^
GO:0006730	one-carbon metabolic process	27	4	0.68	5.88	1^ry^ > 2^ry^
GO:0080167	response to karrikin	44	6	1.11	5.41	1^ry^ > 2^ry^
GO:0009699	phenylpropanoid biosynthetic process	93	12	2.34	5.13	1^ry^ > 2^ry^
GO:0008219	cell death	77	9	1.94	4.64	1^ry^ > 2^ry^
GO:0016998	cell wall macromolecule catabolic process	26	3	0.65	4.62	1^ry^ > 2^ry^
GO:0006555	methionine metabolic process	28	3	0.71	4.23	1^ry^ > 2^ry^
GO:0009611	response to wounding	129	13	3.25	4.00	1^ry^ > 2^ry^
GO:0009787	regulation of abscisic acid-activated signaling pathway	30	3	0.76	3.95	1^ry^ > 2^ry^
GO:0009411	response to UV	53	5	1.33	3.76	1^ry^ > 2^ry^
GO:0009808	lignin metabolic process	85	8	2.14	3.74	1^ry^ > 2^ry^
GO:0000272	polysaccharide catabolic process	64	6	1.61	3.73	1^ry^ > 2^ry^
GO:0009626	plant-type hypersensitive response	44	4	1.11	3.60	1^ry^ > 2^ry^
GO:0009636	response to toxic substance	49	4	1.23	3.25	1^ry^ > 2^ry^
GO:0031408	oxylipin biosynthetic process	51	4	1.28	3.13	1^ry^ > 2^ry^
GO:0055114	oxidation-reduction process	684	36	17.22	2.09	1^ry^ > 2^ry^
Tertiary versus secondary lines						
GO:0006433	prolyl-tRNA aminoacylation	2	2	0.06	33.33	3^ry^ > 2^ry^
GO:0009871	jasmonic acid and ethylene-dependent systemic resistance. Ethylene mediated signaling pathway	5	3	0.16	18.75	3^ry^ > 2^ry^
GO:0009969	xyloglucan biosynthetic process	8	3	0.26	11.54	3^ry^ > 2^ry^
GO:0031640	killing of cells of other organism	11	4	0.35	11.43	3^ry^ > 2^ry^
GO:0010731	protein glutathionylation	7	2	0.22	9.09	3^ry^ > 2^ry^
GO:0010183	pollen tube guidance	14	4	0.45	8.89	3^ry^ > 2^ry^
GO:0006558	L-phenylalanine metabolic process	15	4	0.48	8.33	3^ry^ > 2^ry^
GO:0046487	glyoxylate metabolic process	8	2	0.26	7.69	3^ry^ > 2^ry^
GO:0080092	regulation of pollen tube growth	17	4	0.54	7.41	3^ry^ > 2^ry^
GO:0043650	dicarboxylic acid biosynthetic process	13	3	0.41	7.32	3^ry^ > 2^ry^
GO:0006032	chitin catabolic process	22	5	0.7	7.14	3^ry^ > 2^ry^
GO:0046189	phenol-containing compound biosynthetic process	9	2	0.29	6.90	3^ry^ > 2^ry^
GO:0006949	syncytium formation	9	2	0.29	6.90	3^ry^ > 2^ry^
GO:0080167	response to karrikin	44	8	1.4	5.71	3^ry^ > 2^ry^
GO:0006816	calcium ion transport	11	2	0.35	5.71	3^ry^ > 2^ry^
GO:0009828	plant-type cell wall loosening	11	2	0.35	5.71	3^ry^ > 2^ry^
GO:0009626	plant-type hypersensitive response	44	7	1.4	5.00	3^ry^ > 2^ry^
GO:0000272	polysaccharide catabolic process	64	10	2.04	4.90	3^ry^ > 2^ry^
GO:0072329	monocarboxylic acid catabolic process	42	6	1.34	4.48	3^ry^ > 2^ry^
GO:0006749	glutathione metabolic process	56	8	1.79	4.47	3^ry^ > 2^ry^
GO:0031408	oxylipin biosynthetic process	51	7	1.63	4.29	3^ry^ > 2^ry^
GO:0009718	anthocyanin-containing compound biosynthetic process	23	3	0.73	4.11	3^ry^ > 2^ry^
GO:0019395	fatty acid oxidation	39	5	1.24	4.03	3^ry^ > 2^ry^
GO:0009611	response to wounding	129	15	4.11	3.65	3^ry^ > 2^ry^
GO:0050832	defense response to fungus	102	11	3.25	3.38	3^ry^ > 2^ry^
GO:0009699	phenylpropanoid biosynthetic process	93	10	2.96	3.38	3^ry^ > 2^ry^
GO:0009808	lignin metabolic process	85	9	2.71	3.32	3^ry^ > 2^ry^
GO:0006952	defense response	370	31	11.79	2.63	3^ry^ > 2^ry^
GO:0044248	cellular catabolic process	472	39	15.05	2.59	3^ry^ > 2^ry^
GO:0006979	response to oxidative stress	232	16	7.4	2.16	3^ry^ > 2^ry^
GO:0009617	response to bacterium	201	13	6.41	2.03	3^ry^ > 2^ry^
GO:0055114	oxidation-reduction process	684	44	21.8	2.02	3^ry^ > 2^ry^
GO:0051603	proteolysis involved in cellular protein catabolic process	142	9	4.53	1.99	3^ry^ > 2^ry^
GO:0005975	carbohydrate metabolic process	414	24	13.2	1.82	3^ry^ > 2^ry^
GO:0010038	response to metal ion	393	19	12.53	1.52	3^ry^ > 2^ry^
GO:0006879	cellular iron ion homeostasis	3	2	0.03	66.67	2^ry^ > 3^ry^
GO:0010197	polar nucleus fusion	8	2	0.09	22.22	2^ry^ > 3^ry^
GO:0046274	lignin catabolic process	16	2	0.18	11.11	2^ry^ > 3^ry^
GO:0048509	regulation of meristem development	20	2	0.23	8.70	2^ry^ > 3^ry^
GO:0010051	xylem and phloem pattern formation	24	2	0.27	7.41	2^ry^ > 3^ry^
GO:0000398	mRNA splicing. Via spliceosome	56	4	0.63	6.35	2^ry^ > 3^ry^
GO:0016310	phosphorylation	222	6	2.51	2.39	2^ry^ > 3^ry^
GO:0032774	RNA biosynthetic process	262	7	2.97	2.36	2^ry^ > 3^ry^

*GO.ID*: Gene Ontology Identifiant; *Annot.*: number of annotated Douglas proteins with GO terms in the data set; *Sign.*: number of Douglas proteins in the analysed dataset; *Exp.*: expected number of interesting proteins mapped to the GO term if randomly distributed over all GO terms; *ratio sign./exp*: ratio between significant and expected proteins mapping to the GO term; *Expression*: proteins assigned to specific GO term are over-expressed in 1^ry^ (1^ry^ > 2^ry^), 2^ry^ (2^ry^ > 1^ry^) or 3^ry^ lines (3^ry^ > 2^ry^). Only results with *p*value < 0.05 of the Fisher’s exact test were considered

Eleven significant proteins involved in the networks (PAL2, CCR1, LOX1, DOX1, ERD9, P5CS1, DFR, C17L7.80, OMT1, PKT3, ALDH2B7, *Arabidopsis* annotation) were down-regulated following the first cycle of repetitive somatic embryogenesis and up-regulated following the second cycle. Interestingly, two others were up-regulated following both cycles and identified as endochitinase EP3 and E3 ubiquitin-protein ligase ARI1, both of which are involved in embryo development and pathways such as controlled proteolysis in conifers.

Among the significant proteins, one is related to the RNA-induced silencing complex (RISC), a ribonucleoprotein. RISC is known to incorporate the microRNAs (miRNAs) MIR165A and MIR166A to cleave the corresponding, targeted mRNA. This gene silencing process, participates to the biological process of mRNA splicing and is apparently modulated only during the third cycle of repetitive somatic embryogenesis.

## Discussion

Repetitive somatic embryogenesis enhanced the embryogenic potential of embryogenic lines derived from two Douglas-fir genotypes.

The experiments with two unrelated genotypes (TD17, SD4) with differing embryogenic potential (under the test conditions) provided abundant information about this common “genotype effect”, which has been observed in various conifers [3]. These include *Pinaceae* species such as pines [8] and Douglas-fir [6, 53], and may result from genotype-specific interaction with culture conditions.

Compared to 1^ry^ lines, 2^ry^ lines of both SD4 and TD17 genotypes displayed significantly higher mean embryogenic potential (2155 vs 477 and 240 vs 29 SEs g^− 1^ f.w.; 4.5 and 8.2-fold increases, respectively). Increases in embryogenic potential after a second cycle of somatic embryogenesis (secondary somatic embryogenesis) have been previously recorded in experiments with hybrid larch (1 genotype, 3-fold increase [27]), maritime pine (2 genotypes, 1.4 to 2.3-fold increase [31]) and Douglas-fir (3 genotypes, 1.2 to 4.9-fold increase [6]). This is particularly interesting for “recalcitrant” genotypes with weak embryogenic potential (e.g. TD17), as poor capacity to regenerate selected genotypes via SEs in breeding programs is a major impediment to multivarietal coniferous forestry [7, 8].

In this study, one genotype (SD4) was subjected to a third cycle of somatic embryogenesis (tertiary somatic embryogenesis) to further investigate cumulative effects of repetitive somatic embryogenesis on its embryogenic potential. To our knowledge, this is the first report of the initiation of 3^ry^ coniferous embryogenic lines. Interestingly, two of the three tested 3^ry^ lines (SD4–8-2 and SD4–8-3) exhibited significantly higher embryogenic potential (mean, 4209 SEs g^− 1^ f.w.), and the third 3^ry^ line (SD4–8-1) non-significantly higher embryogenic potential (3344 SEs g^− 1^ f.w.) than the original 2^ry^ line SD4–8 (mean, 2400 SEs g^− 1^ f.w.). We conclude that repetitive somatic embryogenesis enhanced the SD4 genotype’s embryogenic potential. However, the increase in maturation performance observed after the third cycle of somatic embryogenesis was weaker than that observed after the second cycle (1.6- vs 4.5-fold increase on average). Long-term stable embryogenic potential has been demonstrated in some *Pinaceae* species, such as *Larix* spp. [32], whereas ageing effects are highly significant in other species (e.g. *Pinus* spp., [8]). The stable embryogenic potential of proliferating Douglas-fir lines of various physiological ages since initiation (from older 1^ry^ lines to younger 2^ry^ and 3^ry^ lines) observed in this study supports the hypothesis that increases in embryogenic potential following repetitive somatic embryogenesis are mainly due to initiation effects rather than ageing. In conifers, cellular and molecular changes in proliferating lines after one or two successive cycles of somatic embryogenesis have been poorly investigated. To our knowledge, there is only one previous report on physiological and molecular aspects of 1^ry^ and 2^ry^ lines with contrasting embryogenic potential, in *Pinus pinaster* [31]. In this work, we gained new insights, discussed in the following sections, into EMs’ cellular organization (cytology, histology) and molecular physiology after both one and two cycles of repetitive somatic embryogenesis in Douglas-fir (via comparisons of 1^ry^ vs 2^ry^ lines and 2^ry^ vs 3^ry^ lines, respectively).

### Repetitive somatic embryogenesis of Douglas-fir improved immature SEs’ cellular organization

The repetitive somatic embryogenesis markedly improved SEs’ structures, particularly in genotype SD4. Both 1^ry^ and 2^ry^ lines of genotype TD17 produced all types of EMs that we have recently described [6], i.e. polyembryogenic centers, large singulated SEs and small SEs. In contrast, the arrangement of EMs in 1^ry^ and 2^ry^ lines of genotype SD4 markedly differed. The large polyembryogenic centers that predominated in 1^ry^ line SD4 slightly resembled meristemoids that develop during organogenesis or somatic embryogenesis of angiosperms [54, 55], or nodules that form on needle primordia after initiation of somatic embryogenesis from shoot buds of white spruce [23]. Nevertheless, cell arrangements of these structures were closer to arrangements of typical Douglas-fir polyembryogenic centers, with a broad meristem-like part joined to a suspensor part forming a compact cell “package” [6]. However, cells located in this “package” were arranged quite loosely and were not elongated as in polyembryogenic centers of the 2^ry^ and 3^ry^ lines. Moreover, the meristem-like part consisted of a very thin layer of densely cytoplasmic cells, while other cells were vacuolated and accumulated secondary metabolites. Parts that could be regarded as cleavable embryonal heads were rare. These structures were observed in neither 2^ry^ nor 3^ry^ lines, although some structures similar to those observed in SD4 material occurred in addition to well-organized bipolar SEs in lines SD4–2 and SD4–6. In conclusion, repetition of somatic embryogenesis clearly improved EMs’ organization. The increased yields of cotyledonary SEs after maturation may have been due to increases in frequencies of small SEs together with reductions in sizes of polyembryogenic centers (as we observed in 3^ry^ line SD4–8-2). Alternatively, it could be related to the organization of polyembryogenic centers into clusters of distinct embryonal heads of very similar size, as observed in SD4–8-1 and even more strongly in SD4–8-3.

### Repetitive somatic embryogenesis decreased abundance of non-embryogenic cell clusters in Douglas-fir EMs

When EMs are cultivated in clumps, highly mitotically active and growing polyembryogenic centers or singulated early SEs occur on the surface of the clumps, while inner parts usually consist of dying suspensor cells or whole SEs. Such organization resulted in most of the investigated lines of Douglas-fir EMs, especially 3^ry^ lines, having a granular appearance (Additional file 1: Table S1). Similar morphotypes of proliferating embryogenic lines of other *Pinaceae* species, such as *Pinus pinaster*, have been observed, and associated with some morphological traits of embryonal heads and suspensors [56] (and references therein). In *Pinus pinaster* it has been shown that the outer parts of EM clumps have higher embryogenic potential than inner parts. Our past experience with various conifer species indicates that the whitish parts of EMs generally have the highest embryogenic potential, and thus were collected for detailed histological study. In spite of our careful selection of samples, the histological study surprisingly revealed the presence of NECs cells close to the EMs, especially in the 1^ry^ line of genotype TD17 and 2^ry^ lines of SD4 (Additional Table S1). Such NEC clusters, consisting of cells accumulating not only starch grains but also phenolic compounds, have been considered one of the main impediments of in vitro propagation of woody plants [57]. Browning of tissue cultures (as observed in our Douglas-fir lines, Additional file 1: Table S1 and Additional file 2: Figure S1) typically results from oxidation of accumulated phenolic compounds. Browning is reportedly a consequence of high oxidative stress and can eventually cause cell death [58], thereby reducing cultures’ regeneration capacity. We observed cells with phenolic contents either as parts of NEC clusters or as individual loosely arranged groups. In the 1^ry^ SD4 line, cells with phenolic contents were also present in the large meristem-like parts of polyembryogenic centers. Processes resulting in oxidation of phenols in the tissues could have detrimental effects on the embryogenic potential of 1^ry^ lines, especially TD17. Thus, the reduction in numbers of NECs containing phenolic compounds was another desirable effect of the repetitive somatic embryogenesis. We found far fewer NECs in the 2^ry^ lines TD17–1 and SD4–2, and close to zero in 3^ry^ lines induced from SD4–8.

### Proteomic analyses revealed important interactions between proteins and plant growth signaling in “plant development” process during the somatic embryogenesis cycles

We compared protein profiles of both 2^ry^ vs 1^ry^ lines and 3^ry^ vs 2^ry^ lines to identify possible proteomic effects of an additional round of somatic embryogenesis. More proteins were detected during both proteomic analyses (2293–2554, 4813 overall) than in previous analyses based on two-dimensional gel electrophoresis [40, 44] confirming that shotgun-iTRAQ technology has greater potential for both identifying and quantifying proteins. This should be generally valid for all plant species, but the availability of the Douglas-fir transcriptome enabled about 30% more successful protein identifications than use of the *Picea glauca* database. PCA revealed that the type of line (1^ry^, 2^ry^, 3^ry^) accounted for most of the total observed variance in expression patterns of this large set of identified proteins, in both genotypes.

No significant differences in total protein content among the compared embryogenic lines were detected. However, the expression of substantial numbers of proteins was affected by the somatic embryogenesis cycles (162 and 228 were significantly up- or down-regulated following the second and third cycles, respectively). Functional analysis showed that most of these significant proteins are mainly involved in metabolic and cellular processes. It is well known that embryogenic competence is accompanied in plants, including conifers (reviewed in [9]) by active metabolic changes and developmental processes [59] as well as cellular reorganizations [36, 60]. Interestingly, we observed a general “down-regulation” of most of a specific set of significant proteins after the second cycle, and a general “up-regulation” of most of another set (including only 33 common proteins of both set) after the third cycle. Thus, specific expression patterns of significant proteins are apparently established after each somatic embryogenesis cycle in Douglas-fir, at least under our test conditions. It is suggested that induction of somatic embryogenesis promotes large, genome-wide changes in gene expression patterns, possibly through activation of chromatin modifiers or other epigenetic regulators. Such global changes in gene co-expression have been reported in various conifers, such as *Picea abies* at the beginning of embryogenesis [61, 62], and *Pinus pinaster* in transitions between stages of embryo development induced by regulatory signal. The latter study highlighted several epigenetic regulation mechanisms involved in stage-to-stage transitions.

Biological processes associated with significant proteins were identified by both GO analyses (Table 3, Additional Table S4 and Fig. 7) and screening against bibliographic data. The resulting networks, showing connections between significant proteins according to their involvement in biological processes and/or interactions with regulatory factors or induction signals, are presented in Fig. 8. Interpretation of proteomic results based on protein networks is a powerful approach, but has several limitations. First, it only highlights connected proteins in the networks. Second, the bibliographic database used is specific to *Arabidopsis thaliana*, so resulting networks inevitably miss important elements of woody, perennial species’ networks. Third, embryo patterning is still a poorly understood and complex process involving a regulated network of at least 300–450 genes [9].

The discussion of significant proteins according to assigned biological processes and functional categories could be complicated by the possible involvement of proteins in several pathways. Therefore, in four sections we discuss the proteomic changes associated with the somatic embryogenesis cycles affecting “plant development”, “proteolysis”, “signaling by growth regulators and polyphenols” and “stress and redox responses”.

#### Proteomic differences between secondary and primary embryogenic lines

##### Plant development

Proteomic studies of embryogenesis classically reported an increase of the primary metabolism either during maturation, or between lines with variable embryogenic potential [63]. Thus our results, showing that more than 50% of the significant proteins are involved in primary metabolism are consistent with previous studies. Our GO analyses and histological results also confirmed the importance of programmed cell death (PCD) during various steps of somatic embryogenesis, including differentiation of proliferating early embryos in EMs into cotyledonary SEs [64, 65]. Also, during the enrichment analyzes of the GOs, this process emerged, in agreement with our histological results. Few proteins are directly related to PCD, or indirectly related via interaction with flavonoids, jasmonic acid, oxidative stress and proteolysis (Table 3 and Additional file 4: Figure S3B,C,E,F). Thus, strong interactions between these protagonists are cited in the literature, as discussed in the following sections. ERD9, a glutathione S-transferase (GST) that is down-regulated in 2^ry^ lines, is directly associated with the biological process “Plant development”, and plays well-known roles in oxidative stress regulation in plants [66], as well as in somatic embryogenesis [67]. Among the up-regulated proteins in 2^ry^ lines, the putative methyltransferase DDB could be a key mediator of increases in embryogenic potential through its involvement in epigenetic mechanisms that are known to promote embryonic development [63]. More specifically, a high level of methylation during embryogenesis has been associated with chromatin remodeling, allowing the expression of genes involved in embryogenesis [62]. Accordingly, an E3 ubiquitin protein ligase (ARI1) was up-regulated in 2^ry^ lines. Ubiquitin protein ligases have known association with activation of chromatin modifiers called ubiquitin−/small ubiquitin-related modifier (SUMO)-conjugating genes, resulting in global modifications of gene expression (see [44] and references therein). EP3 chitinase was also up-regulated in the lines produced by both second and third somatic embryogenesis. This is consistent with indications that chitinases play important non-defensive roles in SEs development, and both carbon and nitrogen metabolism in embryogenic lines [12, 42, 68], as well as the development of somatic and zygotic embryos [69]. Chitinases are also associated with PCD in plants [69].

##### Proteolysis

The observed increases in embryogenic potential were apparently accompanied by significant degradation and recycling of proteins, while the total protein content remained roughly constant (Additional Tables S2 and S3). These conclusions are supported by the GO term enrichments (Fig. 7) and presence of cathepsin B-like proteins, probable E3 ubiquitin-protein ligase ARI1 and serine carboxypeptidase in the sets of significantly up-regulated proteins in both the second and third somatic embryogenesis cycles (Additional file 7). The findings also emphasize the importance of proteolysis in increasing embryogenic potential [62]. Cathepsin B-like protein, a cysteine ​​protease, plays a key in degradation of target proteins and participates in embryogenesis, plant defense and PCD [70]. Its presence in embryogenic calli has been associated with involvement in maintenance of pluripotency and cell reprogramming these tissues [71]. The probable E3 ubiquitin-protein ligase ARI1 is also involved in protein recycling, as part of the ubiquitin/26S proteasome complex. Substantial support has been obtained for regarding ubiquitin protein ligase as robust marker of correct embryo development in *Pinus pinaster* [12]. The last protein identified in this protein renewal pathway is an ABA-inducible serine carboxypeptidase, involved in secondary metabolism and stress responses [72]. These two biological processes were modified during induction according to the protein networks and are discussed in a following section. A recent study revealed that serine carboxypeptidase has positive effects in embryogenesis, and more specifically polyembryogenesis induction [73]. Finally, expression of this protein is regulated by pathogen response pathways and jasmonic acid [74], a plant growth regulator that connects several proteins in the significant protein network (Fig. 8).

##### Signaling by plant growth regulators and polyphenols

Plant signaling networks are crucial for the control of every embryogenesis stage, from EMs’ proliferation through maturation to germination. Accordingly, the proteomic comparisons demonstrated the involvement of several signaling compounds in 2^ry^ somatic embryogenesis, by highlighting their interactions with various significant hormone-responsive proteins. Jasmonic acid was the one with most connections. All of the associated proteins except one (L-galactono-1,4-lactone dehydrogenase) were more strongly expressed in 1^ry^ lines than in 2^ry^ lines. Several studies have detected positive effects of jasmonic acid on maturation of SEs [75]. This regulator, like ABA and salicylic acid, is mainly linked to biotic and abiotic stresses [76] in the pre-embryonic stage of development, as illustrated by connections of the protein network. Proteins connected with jasmonic acid are also linked to GO terms related to the synthesis of flavonoids, anthocyanidins and/or anthocynins, all of which are secondary metabolites that act as signals in cell development functions [77]. Overabundance of such metabolites has been shown to hinder embryogenesis as they appear to induce formation of unpolarized or irregular structures of NECs cells [78]. Flavonoid-related proteins were down-regulated in 2^ry^ lines, which may explain their higher embryogenic potential. These flavonoids play important roles in the cells since they have several links with oxidative stress responses [79, 80]. Flavonoids are polyphenols (compounds derived from phenylpropanoids), and phenylalanine ammonia-lyase (PAL) is the first enzyme involved in their committed synthesis from the amino acid phenylalanine. Flavonoids are polyphenols (compounds derived from phenylpropanoids), and phenylalanine ammonia-lyase (PAL) is the first enzyme involved in their committed synthesis from the amino acid phenylalanine. Two PAL-like proteins were strongly expressed in the 1^ry^ lines, indicating activation of the phenylpropanoid synthesis pathway and phenylalanine metabolism, which could explain their relatively high contents of polyphenols (Fig. 4c,h) and brownish color.

##### Stress and redox responses

Oxidative stress, one of various stresses affecting cell cultures, appears to be a key factor for somatic embryogenesis [67, 81], which seems to follow substantial production of ROS (Reactive Oxygen Species). These molecules may play antagonistic roles in cell development depending on their concentrations. For example, overproduction of ROS impairs embryogenic differentiation [82], but prevention of their production also inhibits development of SEs [63]. This is because they are toxic to cells, but act as signaling molecules in responses to both biotic and abiotic stresses. As ROS are potentially lethal to cells, their presence induces synthesis of numerous proteins that regulate cellular redox systems. ROS are involved in PCD activation [83], and thus participate in the normal development of embryos [62]. The proteins involved in oxidative stress responses may vary, depending on the stage of embryogenesis. For example, catalase is reportedly more abundant during proliferation and organogenesis than at other stages [81], and more strongly expressed in non-embryogenic calli than in embryonal masses [78]. Accordingly, we found that catalase isozyme 1-like (CAT3) protein, a PCD effector, was more strongly expressed in the 1^ry^ lines than in the more embryogenic 2^ry^ lines. In contrast, several peroxidases (which have well-established involvement in defense mechanisms, such as oxidative stress responses [43, 81], as well as proliferation and maturation) were significantly up-regulated in the 2^ry^ lines. An NAD(P)-binding Rossmann-fold protein, involved in redox reactions, was also up-regulated in 2^ry^ and 3^ry^ lines, relative to 1^ry^ lines. In contrast, other proteins possibly involved in ROS responses were down-regulated in 2^ry^ lines, for example the GST ERD9 and PAL-like protein. Relationships have been established between the latter enzyme and both flavonoids and polyphenols, as previously discussed. GSTs are a multigene family that may be involved in auxin responses, production of secondary metabolites (anthocyanins), and confer antioxidant activity to flavonoids [66].

In conclusion, characteristic biological processes of embryogenesis, such as “plant development”, “defense” and “stress” (especially oxidative) “metabolism” and “proteolysis” were affected during proliferation by repeated somatic embryogenesis cycles. Moreover, the major result is the identification of numerous proteins that interact with flavonoids and associated secondary metabolites were down-regulated following the second somatic embryogenesis cycle.

#### Proteomic differences between tertiary and secondary embryogenic lines

The 3^ry^ lines had greater embryogenic potential than the 1^ry^ and 2^ry^ lines, and comparison of the 2^ry^ and 3^ry^ lines’ proteomes revealed activation of the same biological processes following the second and third cycles of somatic embryogenesis. Thus, the biological processes “Plant development”, “Proteolysis”, “Stress/water stress”, “Defense response” and “Growth regulators” also appear in the networks derived from the significant proteins associated with the third cycle (Fig. 8b). However, the proteins were often different from those associated with the second cycle, only 33 proteins were present in both sets of “significant” proteins (i.e. proteins significantly differentially expressed between 2^ry^ and 1^ry^ EMs, and between 2^ry^ and 3^ry^ EMs) among 390 identified significant proteins in total. Furthermore, only 12 were either up-regulated or down-regulated following both cycles. These findings show that embryogenic induction is a complex process that changes the cells’ proteomic composition. Induction is triggered by transfer of the cotyledonary SEs to an appropriate culture medium containing auxin. The repeated intake of auxin during the three somatic embryogenesis cycles presumably affected the EMs, since auxin interacts (*inter alia*) with flavonoids, ABA, jasmonic acid and salicylic acid [84]. Salicylic acid, which participates in regulation of plant growth, as well as both biotic and abiotic stresses responses [85], also interacts strongly with flavonoids [86] and jasmonic acid. Thus, these changes inevitably affected the proteome composition of 3^ry^ lines and then expression of genes that are sensitive to, or involved in metabolism of, key signaling compounds (Fig. 8b) that regulate cell growth and differentiation [79, 87, 88]. The high number of ABA-sensitive proteins up-regulated in the 3^ry^ line, and histological results, suggest that the corresponding EMs were in a more advanced stage of embryogenesis. The 3^ry^ lines seemed to be composed of EMs with better-organized embryonal heads, probably with more divisions.

In summary, the embryogenic character is acquired through activation of mechanisms related to stress and defense responses, interactively with growth regulators, and shifts in expression of proteins associated with indirectly involved biological processes of “Metabolic process” and “Plant development”.

## Conclusions

Repetitive somatic embryogenesis improved the SEs’ structure by increasing frequencies of small SEs and reducing sizes of polyembryogenic centers. Each cycle of embryogenesis induced modifications in the expression of proteins connected to biological processes known to be related to somatic embryogenesis, but lacking previously known association with EMs (defense and stress responses, and various plant development metabolic, and proteolytic processes). The innovative use of protein networks in the proteomic analysis had been very conclusive. It provided valuable information, revealing the general down-regulation and up-regulation of significant proteins following the first and second somatic embryogenesis cycles, respectively. In both cases, interactions with various plant growth signaling agents (flavonoids and associated compounds, jasmonic acid, ABA, auxin, salicylic acid) were major elements of the shifts, showing the ability of cells to use different protein regulatory pathways to increase embryogenic potential, and resulting in more suitable SEs for maturation. Overall, this first report of cellular and molecular changes in EMs after two successive cycles of somatic embryogenesis in conifers generally, and Douglas-fir specifically, should enhance understanding of the increases in embryogenic potential of 2^ry^ 3^ry^ embryogenic lines.

These findings could also help with practical application. A number of laboratories now have the problem of deterioration in the quality of elite somatic embryogenic lines due to extensive subculture. The methods described in this paper could be of great benefit to reinvigorate these valuable proven production clones.

## Additional files

Additional file 1: **Table S1.** A summary of macromorphological (EM colour and morphotype) and histo-cytological traits (occurrence of polyembryogenic centers (PECs), singulated SEs and NECs) of 1^ry^ (SD4, TD17), 2^ry^ (SD4–2, SD4–6, SD4–8; TD17–1) and 3^ry^ (SD4–8-1, SD4–8-2, SD4–8-3) embryogenic lines of Douglas-fir. (DOCX 16 kb)

Additional file 2: **Figure S1**. Macroscopic aspect of embryonal masses (EMs) from two Douglas-fir genotypes (SD4, TD17). These embryonal masses were obtained after 1^ry^ somatic embryogenesis from zygotic embryo and two cycles (2^ry^ and 3^ry^) of repetitive somatic embryogenesis from somatic embryos. Note the granular aspect of most embryonal masses indicating the occurrence of large polyembryogenic centers (arrowheads) and/or singulated early somatic embryos that are sometimes protruding from the embryonal masses surface. (DOCX 687 kb)

Additional file 3: **Figure S2.** Structure of embryonal masses from primary and secondary lines of genotype TD17. **A** / TD17; **B** / TD17–1. Trypan blue staining of squashes of fresh EMs; m – meristem of polyembryogenic centers, s – suspensor. Scale bar = 200 μm. (DOCX 178 kb)

Additional file 4: **Figure S3.** Histology of non-embryogenic cells (NECs) clusters from 1^ry^ and/or 2^ry^ lines of genotypes TD17 and SD4. **A** / Histology of TD17 groups of loosely arranged NECs (arrow) in the vicinity of small somatic embryos (arrowhead); **B** / histology of TD17 compact NEC cluster accumulating phenolics (blue and brown cells, arrowheads) and starch (arrow); **C** / Lugol staining of TD17 NEC cluster showing starch (arrows) and phenolic compounds (small dark granules marked with arrowhead); **D** / TD17–1 NEC cluster with dividing cells (arrowheads); **E** /SD4–2 NEC cluster (arrow) within polyembryogenic center besides well-arranged embryonal heads (EH), note phenolic content (in dark blue-grey) of cells separating NEC from suspensor cells (in light blue); **F** / SD4–6 meristemoid-like NEC cluster; note phenolic content of cells between two meristemoid-like structures (in blue-grey). Scale bar: A, E = 200 μm; B, F = 100 μm; C, D = 50 μm. (DOCX 417 kb)

Additional file 5: **Table S2.** Total protein content (mean ± SD, *n* = 4) in proliferating 1^ry^ and 2^ry^ embryogenic lines of Douglas-fir. (DOCX 45 kb)

Additional file 6: **Table S3.** Total protein content (mean ± SD, n = 4) in proliferating 2^ry^ and 3^ry^ embryogenic lines of Douglas-fir. (DOCX 45 kb)

Additional file 7: Differentially expressed proteins in 2^ry^ vs 1^ry^ and 3^ry^ vs 2^ry^ embryogenic lines of two Douglas-fir genotypes. (XLSX 2224 kb)

Additional file 8: **Table S4**. Functional classification according to gene ontology (GO) of significant proteins identified after two cycles of repetitive somatic embryogenesis in Douglas-fir. (DOCX 72 kb)

## Abbreviations

1^ry^: Primary
2^ry^: Secondary
3^ry^: Tertiary
ABA: Abscisic acid
d.w.: Dry weight
EMs: Embryonal masses
f.w.: Fresh weight
GO: Gene ontology
LC-MS/MS: Liquid chromatography coupled to tandem mass spectrometry
NECs: Non-embryogenic cells
PCD: Programmed cell death
ROS: Reactive oxygen species
SEs: Somatic embryos
SNEA: Sub-network enrichment analysis
