# Supplementary material for: Repetitive somatic embryogenesis induced cytological and proteomic changes in embryogenic lines of Pseudotsuga menziesii [Mirb.]
Source: BMC Plant Biol. 2018 Aug 10;18:164. doi: 10.1186/s12870-018-1337-y (PMC6086078; doi:10.1186/s12870-018-1337-y)
Supplement: Supplementary file 1 — Table S1. A summary of macromorphological (EM colour and morphotype) and histo-cytological traits (occurrence of polyembryogenic centers (PECs), singulated SEs and NECs) of 1ry (SD4, TD17), 2ry (SD4–2, SD4–6, SD4–8; TD17–1) and 3ry (SD4–8-1, SD4–8-2, SD4–8-3) embryogenic lines of Douglas-fir. (DOCX 16 kb) [file 12870_2018_1337_MOESM1_ESM.docx]

**Additional file Table S1.** A summary of macromorphological (EM colour and morphotype) and histo-cytological traits (occurrence of polyembryogenic centres (PECs), singulated SEs and NECs) of 1^ry^ (SD4, TD17), 2^ry^ (SD4-2, SD4-6, SD4-8; TD17-1) and 3^ry^ (SD4-8-1, SD4-8-2, SD4-8-3) embryogenic lines of Douglas-fir.

| **Trait** | **Primary lines** | **Secondary lines** | **Tertiary lines** |
| --- | --- | --- | --- |
| EM colour | Mostly yellow/brown, or pink, some whitish parts | Mixture of yellowish and whitish parts | Mostly whitish, some yellowish parts |
| EM morphotype | Granular with few protruding SEs (SD4 > TD17) | Smooth (SD4-8) to granular (SD4-6) with frequent protruding SEs (TD17-1, SD4-2) | Granular with frequent protruding SEs (SD4-8-1, SD4-8-3 > SD4-8-2) |
| Occurrence of PECs | + (moderately frequent, large PECs)  **TD17**: frequent compact PECs  **SD4**: huge structures resembling PEC (meristemoids/nodules) but few elongated suspensor cells and starch and/or phenolics accumulating in some vacuolated cells. Rare parts considered as cleavable embryonal heads | ++ (frequent PECs of small to large size)  **TD17-1**: frequent compact PECs  **SD4-2**: mostly PECs with distinct embryonal heads and a few structures similar to meristemoids  **SD4-6**: highly frequent small PECs with noticeable embryonal heads + rare PECs of huge size and a frequent structure with meristemoid aspect  **SD4-8**: only smaller PECs with lower organization | +++ (highly frequent PECs, more fragmented into individual embryonal heads)  **SD4-8-1**: PECs organized into clusters of distinct embryonal heads of similar size  **SD4-8-2**: high amount of PECs of reduced size with some signs of disintegration of both meristems and suspensor  **SD4-8-3**: PECs organized into clusters of well-organized embryonal heads of similar size joined to dense suspensors |
| Occurrence of singulated SEs | + (rare small SEs in close proximity to remnants of suspensor cells or disintegrating SE, low frequency of large singulated SEs with well-organized embryonal heads)  **TD17**: rare small SEs, higher frequency of large singulated SEs  **SD4**: rare small to large SEs | ++ (rare to highly frequent small SEs close to suspensors)  **TD17-1**: rare small SEs, higher frequency of large singulated SEs  **SD4-2**: small SEs located within suspensors  **SD4-6**: highly frequent small SEs  **SD4-8**: frequent small SEs | +++ (highly frequent small to large SEs close to suspensors)  **SD4-8-1**: frequent clusters of large SEs with distinct embryonal heads and smaller SEs occurring within suspensors.  **SD4-8-2**: increased frequency of small SEs  **SD4-8-3**: frequent large SEs and smaller SEs within or in the vicinity of suspensors |
| Occurrence of NEC clusters | +++ (frequent small to large NEC clusters close to SE and/or within dead material)  **TD17**: NECs in close proximity to EMs organized as compact clusters or groups of loosely arranged vacuolated cells of irregular shape accumulating starch grains and/or phenolics  **SD4**: no organized individual clusters of NECs, some parts of huge PEC-like structures resemble NEC by chaotic organization of cells and accumulation of starch grains and/or phenolic compounds | ++ (moderately frequent small to large NEC clusters close to SE and/or within dead material)  **TD17-1**: small NEC pieces, mostly within dead material  **SD4-2**: some parts of PEC-like structures composed of vacuolated NECs containing starch grains and/or phenolics  **SD4-6**: frequent small to large and compact NEC clusters (meristemoids surrounded by groups of cells with high phenolic content)  **SD4-8**: NEC clusters of vacuolated cells containing starch grains and/or phenolics | + (very low frequency of NEC clusters of smaller size) |
