# Supplementary figures and images for: Repetitive somatic embryogenesis induced cytological and proteomic changes in embryogenic lines of Pseudotsuga menziesii [Mirb.]
Source: BMC Plant Biol. 2018 Aug 10;18:164. doi: 10.1186/s12870-018-1337-y (PMC6086078; doi:10.1186/s12870-018-1337-y)

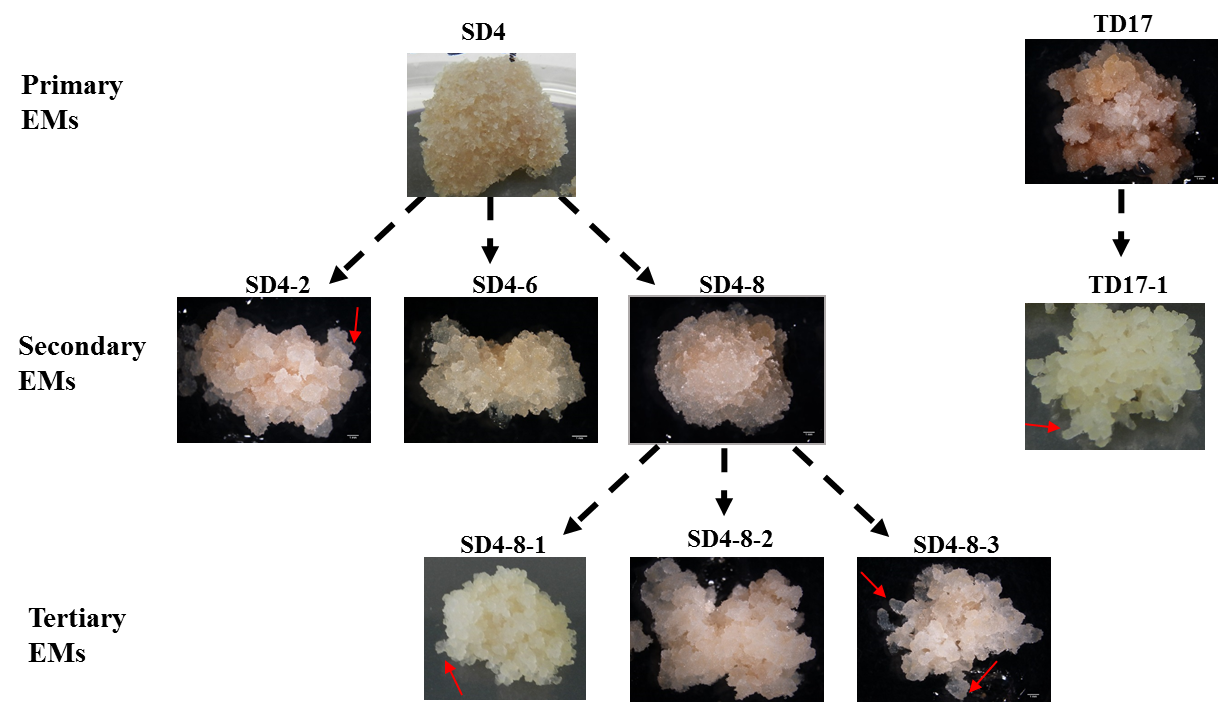


**Additional file Fig S1**.

Supplement: Supplementary file 2 — Figure S1. Macroscopic aspect of embryonal masses (EMs) from two Douglas-fir genotypes (SD4, TD17). These embryonal masses were obtained after 1ry somatic embryogenesis from zygotic embryo and two cycles (2ry and 3ry) of repetitive somatic embryogenesis from somatic embryos. Note the granular aspect of most embryonal masses indicating the occurrence of large polyembryogenic centers (arrowheads) and/or singulated early somatic embryos that are sometimes protruding from the embryonal masses surface. (DOCX 687 kb) [file 12870_2018_1337_MOESM2_ESM.docx]

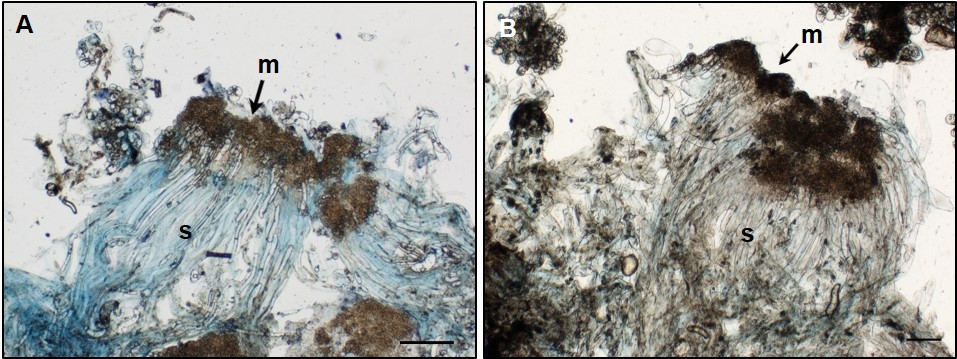


**Additional file Fig S2.**

Supplement: Supplementary file 3 — Figure S2. Structure of embryonal masses from primary and secondary lines of genotype TD17. A / TD17; B / TD17–1. Trypan blue staining of squashes of fresh EMs; m – meristem of polyembryogenic centers, s – suspensor. Scale bar = 200 μm. (DOCX 178 kb) [file 12870_2018_1337_MOESM3_ESM.docx]

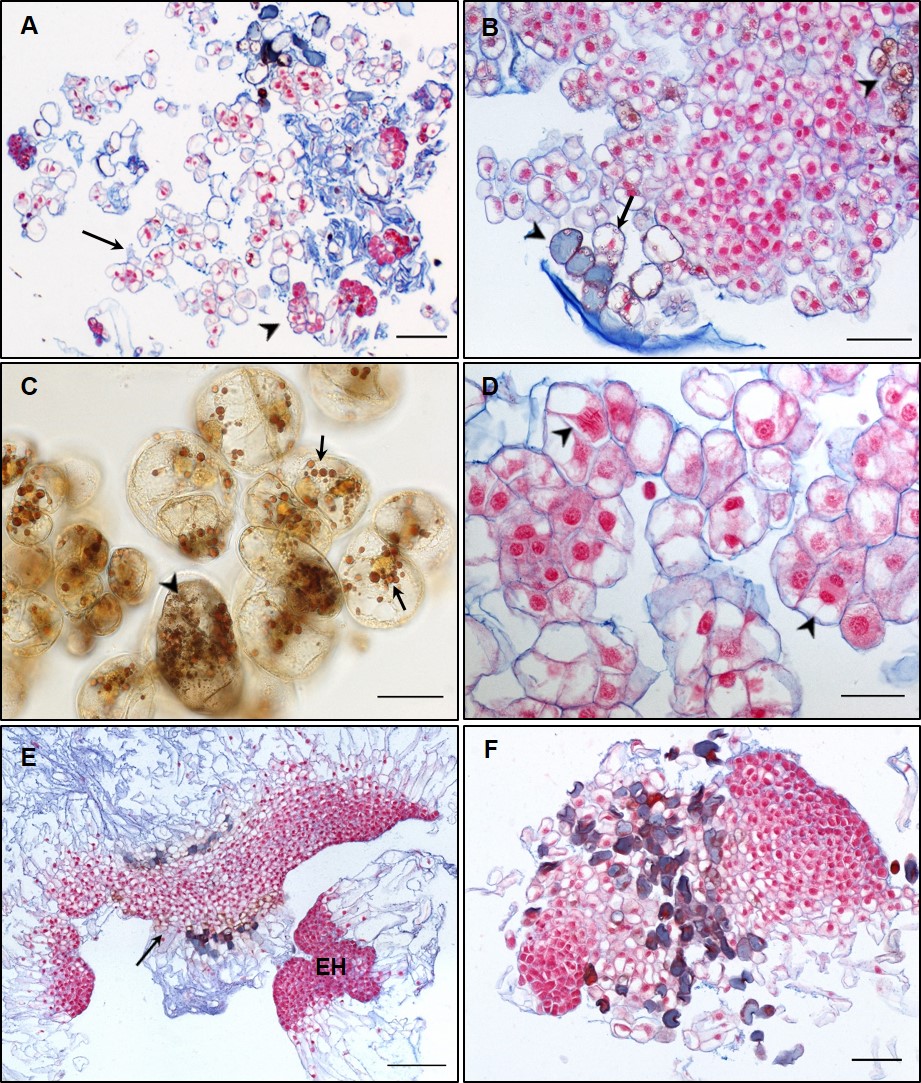


**Additional file Fig S3.**

Supplement: Supplementary file 4 — Figure S3. Histology of non-embryogenic cells (NECs) clusters from 1ry and/or 2ry lines of genotypes TD17 and SD4. A / Histology of TD17 groups of loosely arranged NECs (arrow) in the vicinity of small somatic embryos (arrowhead); B / histology of TD17 compact NEC cluster accumulating phenolics (blue and brown cells, arrowheads) and starch (arrow); C / Lugol staining of TD17 NEC cluster showing starch (arrows) and phenolic compounds (small dark granules marked with arrowhead); D / TD17–1 NEC cluster with dividing cells (arrowheads); E /SD4–2 NEC cluster (arrow) within polyembryogenic center besides well-arranged embryonal heads (EH), note phenolic content (in dark blue-grey) of cells separating NEC from suspensor cells (in light blue); F / SD4–6 meristemoid-like NEC cluster; note phenolic content of cells between two meristemoid-like structures (in blue-grey). Scale bar: A, E = 200 μm; B, F = 100 μm; C, D = 50 μm. (DOCX 417 kb) [file 12870_2018_1337_MOESM4_ESM.docx]
