# Supplementary material for: Repetitive somatic embryogenesis induced cytological and proteomic changes in embryogenic lines of Pseudotsuga menziesii [Mirb.]
Source: BMC Plant Biol. 2018 Aug 10;18:164. doi: 10.1186/s12870-018-1337-y (PMC6086078; doi:10.1186/s12870-018-1337-y)
Supplement: Supplementary file 5 — Table S2. Total protein content (mean ± SD, n = 4) in proliferating 1ry and 2ry embryogenic lines of Douglas-fir. (DOCX 45 kb) [file 12870_2018_1337_MOESM5_ESM.docx]

**Additional file Table S2.** Total protein content (mean ± SD, n=4) in proliferating 1^ry^ and 2^ry^ embryogenic lines of Douglas-fir.

| Line | µg protein mg^-1^ d.w.* |
| --- | --- |
| primary |  |
| TD17 | 107.3 ± 13.7 ^α^ |
| SD4 | 107.1 ± 22.4 ^αβ^ |
| secondary |  |
| TD17-1 | 89.3 ± 12.4^α^ |
| SD4-2 | 131.2 ± 12.6^α^ |
| SD4-6 | 123.9 ± 7.0 ^α^ |
| SD4-8 | 91.5 ± 16.0 ^β^ |

*Letters represent statistical groups defined by the multiple comparisons of means (*P<0.05*).
