# Supplementary material for: Repetitive somatic embryogenesis induced cytological and proteomic changes in embryogenic lines of Pseudotsuga menziesii [Mirb.]
Source: BMC Plant Biol. 2018 Aug 10;18:164. doi: 10.1186/s12870-018-1337-y (PMC6086078; doi:10.1186/s12870-018-1337-y)
Supplement: Supplementary file 6 — Table S3. Total protein content (mean ± SD, n = 4) in proliferating 2ry and 3ry embryogenic lines of Douglas-fir. (DOCX 45 kb) [file 12870_2018_1337_MOESM6_ESM.docx]

**Additional file Table S3.** Total protein content (mean ± SD, n=4) in proliferating 2^ry^ and 3^ry^ embryogenic lines of Douglas-fir.

| Line | µg protein mg^-1^ d.w.* |
| --- | --- |
| secondary |  |
| SD4-8 | 110.8 ± 29.8 ^α^ |
| tertiary |  |
| SD4-8-1 | 153.7 ± 46.1 ^α^ |
| SD4-8-2 | 134.4 ± 29.1 ^α^ |
| SD4-8-3 | 124.5 ± 27.8 ^α^ |

*Letters represent statistical groups defined by the multiple comparisons of means (*P<0.05*)*.*
