# Supplementary material for: Repetitive somatic embryogenesis induced cytological and proteomic changes in embryogenic lines of Pseudotsuga menziesii [Mirb.]
Source: BMC Plant Biol. 2018 Aug 10;18:164. doi: 10.1186/s12870-018-1337-y (PMC6086078; doi:10.1186/s12870-018-1337-y)
Supplement: Supplementary file 8 — Table S4. Functional classification according to gene ontology (GO) of significant proteins identified after two cycles of repetitive somatic embryogenesis in Douglas-fir. (DOCX 72 kb) [file 12870_2018_1337_MOESM8_ESM.docx]

**Additional file Table S4**. Functional classification according to gene ontology (GO) of significant proteins identified after two cycles of repetitive somatic embryogenesis in Douglas-fir.

| **Secondary versus primary lines** | |  | |  |  | |
| --- | --- | --- | --- | --- | --- | --- |
| **Functional class** | | **1^ry^ lines** | |  | **2^ry^ lines** | |
|  |  | **no.** | **%** |  | **no.** | **%** |
| **Response to stimulus (GO:0050896)** | | **6** | **7.8** |  | **2** | **4.5** |
| **Developmental process (GO:0032502)** | | **0** | **0** |  | **1** | **2.3** |
| **Cellular process (GO:0009987)** | | **16** | **20.8** |  | **17** | **38.6** |
| **Metabolic process (GO:0008152)** | | **45** | **58.4** |  | **19** | **43.2** |
|  | *Biosynthetic process (GO:0009058)* | *8* | *12.7* |  | *4* | *9.3* |
|  | *Nitrogen compound metabolic process (GO:0006807)* | *5* | *7.9* |  | *9* | *20.9* |
|  | *Phosphate-containing compound metabolic process (GO:0006796)* | *3* | *4.8* |  | *3* | *7* |
|  | *Generation of precursor metabolites and energy (GO:0006091)* | *3* | *4.8* |  | *1* | *2.3* |
|  | *Catabolic process (GO:0009056)* | *7* | *11.1* |  | *9* | *20.9* |
|  | *Primary metabolic process (GO:0044238)* | *32* | *50.8* |  | *16* | *37.2* |
|  | *Coenzyme metabolic process (GO:0006732)* | *1* | *1.6* |  | *1* | *2.3* |
|  | *Secondary metabolic process (GO:0019748)* | *4* | *6.3* |  | *0* | *0* |
| **Biological regulation (GO:0065007)** | | **2** | **2.6** |  | **1** | **2.3** |
| **Cellular component organization or biogenesis (GO:0071840)** | | **3** | **3.9** |  | **3** | **6.8** |
| **Localization (GO:0051179)** | | **5** | **6.5** |  | **1** | **2.3** |

| **Tertiary versus secondary lines** | | | | | | |
| --- | --- | --- | --- | --- | --- | --- |
| **Functional class** | | **2^ry^ line** | |  | **3^ry^ lines** | |
|  |  | **no.** | **%** |  | **no.** | **%** |
| **Response to stimulus (GO:0050896)** | | **5** | **7.2** |  | **7** | **7.4** |
| **Developmental process (GO:0032502)** | | **0** | **0** |  | **1** | **1.1** |
| **Cellular process (GO:0009987)** | | **21** | **30.4** |  | **29** | **31** |
|  | *Cell cycle (GO:0007049)* | *2* | *40* |  | *1* | *100* |
|  | *Cytokinesis (GO:0000910)* | *1* | *20* |  | *0* | *0* |
|  | *Cell cycle (GO:0007049)* | *2* | *40* |  | *0* | *0* |
| **Metabolic process (GO:0008152)** | | **26** | **37.7** |  | **49** | **52** |
|  | *Phosphate-containing compound metabolic process (GO:0006796)* | *6* | *10.2* |  | *3* | *3.3* |
|  | *Biosynthetic process (GO:0009058)* | *6* | *10.2* |  | *6* | *6.7* |
|  | *Vitamin metabolic process (GO:0006766)* | *1* | *1.7* |  | *2* | *2.2* |
|  | *Primary metabolic process (GO:0044238)* | *25* | *42.4* |  | *37* | *41* |
|  | *Catabolic process (GO:0009056)* | *4* | *6.8* |  | *20* | *22* |
|  | *Sulfur compound metabolic process (GO:0006790)* | *0* | *0* |  | *2* | *2.2* |
|  | *Coenzyme metabolic process (GO:0006732)* | *1* | *1.7* |  | *5* | *5.6* |
|  | *Nitrogen compound metabolic process (GO:0006807)* | *14* | *23.7* |  | *9* | *10* |
|  | *Generation of precursor metabolites and energy (GO:0006091)* | *2* | *3.4* |  | *2* | *2.2* |
|  | *Secondary metabolic process (GO:0019748)* | *0* | *0* |  | *4* | *4.4* |
| **Biological regulation (GO:0065007)** | | **5** | **7.2** |  | **2** | **2.1** |
| **Cellular component organization or biogenesis (GO:0071840)** | | **7** | **10.1** |  | **3** | **3.2** |
| **Localization (GO:0051179)** | | **5** | **7.2** |  | **3** | **3.2** |

For each GO. the total number (no.) of proteins is indicated in the first column and the corresponding percentage in the second column.
